# A Coccidia-Specific Phosphate Transporter Is Essential for the Growth of Toxoplasma gondii Parasites

**DOI:** 10.1128/spectrum.02186-22

**Published:** 2022-09-12

**Authors:** Jianmin Cui, Xuke Yang, Jichao Yang, Ruilian Jia, Yaoyu Feng, Bang Shen

**Affiliations:** a State Key Laboratory of Agricultural Microbiology, College of Veterinary Medicine, Huazhong Agricultural University, Wuhan, Hubei Province, People’s Republic of China; b Key Laboratory of Preventive Medicine in Hubei Province, Huazhong Agricultural University, Wuhan, Hubei Province, People’s Republic of China; c Hubei Hongshan Laboratory, Wuhan, Hubei Province, People’s Republic of China; d Center for Emerging and Zoonotic Diseases, College of Veterinary Medicine, South China Agricultural University, Guangzhou, People’s Republic of China; Hubei University of Medicine

**Keywords:** phosphate transporter, coccidia, ATP scavenge, *Toxoplasma*

## Abstract

Toxoplasma gondii is an obligate intracellular parasite that acquires all necessary nutrients from the hosts, but the exact nutrient acquisition mechanisms are poorly understood. Here, we identified three putative phosphate transporters in T. gondii. TgPiT and TgPT2 are mainly on the plasma membrane, whereas TgmPT is localized to the mitochondrion. TgPiT and TgmPT are widely present and conserved in apicomplexan parasites that include *Plasmodium* and *Eimeria* species. Nonetheless, they are dispensable for the growth and virulence of *Toxoplasma*. TgPT2, on the other hand, is restricted to coccidia parasites and is essential for *Toxoplasma* survival. TgPT2 depletion led to reduced motility and invasion, as well as growth arrest of the parasites both *in vitro* and *in vivo*. Both TgPiT and TgPT2 have phosphate transport activities and contribute to parasites’ inorganic phosphate (P_i_) absorption. Interestingly, the P_i_ importing activity of *Toxoplasma* parasites could be competitively inhibited by ATP and AMP. Furthermore, direct uptake of ^32^P-ATP was also observed, indicating the parasites’ ability to scavenge host ATP. Nonetheless, ATP/AMP import is not mediated by TgPiT or TgPT2, suggesting additional mechanisms. Together, these results show the complex pathways of phosphate transport in *Toxoplasma*, and TgPT2 is a potential target for antitoxoplasmic intervention design due to its essential role in parasite growth.

**IMPORTANCE** To grow and survive within host cells, *Toxoplasma* must scavenge necessary nutrients from hosts to support its parasitism. Transporters located in the plasma membrane of the parasites play critical roles in nutrient acquisition. *Toxoplasma* encodes a large number of transporters, but so far, only a few have been characterized. In this study, we identified two phosphate transporters, TgPiT and TgPT2, to localize to the plasma membrane of *Toxoplasma.* Although both TgPiT and TgPT2 possess phosphate transport activities, only the novel transporter TgPT2 was essential for parasite growth, both *in vitro* and *in vivo*. In addition, TgPT2 and its orthologs are only present in coccidia parasites. As such, TgPT2 represents a potential target for drug design against toxoplasmosis. In addition, our data indicated that *Toxoplasma* can take up ATP and AMP from the environment, providing new insights into the energy metabolism of *Toxoplasma*.

## INTRODUCTION

Toxoplasma gondii is a ubiquitous pathogen belonging to the phylum apicomplexan, which includes many important human and animal parasites such as *Plasmodium*, *Cryptosporidium*, and *Eimeria* species (1, 2). T. gondii is a major foodborne pathogen infecting one-third of the world’s human population (3). Human infections are often asymptomatic in otherwise healthy individuals but can cause severe complications in immunocompromised individuals (4, 5). In addition, if the first infection is acquired during pregnancy, it can cause abortion and serious congenital diseases (6, 7). Limited chemotherapies are currently available, but they are far from ideal due to strong side effects (8).

T. gondii is an obligate intracellular parasite residing in parasitophorous vacuoles (PVs) that are nonfusogenic with the hosts’ endolysosomal system (9). As such, *Toxoplasma* relies on host cells to provide all necessary nutrients for proliferation and survival. Therefore, it has sophisticated strategies to acquire nutrients from host cells, and cross-membrane transport of various molecules is pivotal to parasite growth. While the propagating parasites are enclosed by the PV membrane (PVM), they secrete proteins such as GRA17 and GRA23 to the PVM to form pores that allow the exchange of molecules below the size of 1,900 Da (10, 11). The permeability of the parasites’ plasma membranes is more selective than that of the PVM. Therefore, specific transporters are required for the nutrients in the PV to enter the parasite cytosol. A family of plasma membrane localized amino acid transporters named ApiAT have been identified in *Toxoplasma* and related parasites. Among them, the tyrosine and aromatic amino acid transporter TgApiAT5-3, the arginine transporter TgApiAT1 (also called TgNPT1), and the lysine transporter ApiAT6-1 are shown to be critical for parasite growth and virulence (12–14). Similarly, other transporters involved in the uptake of diverse nutrients, such as sugars, nucleosides, and ions are also described or reported (15–17). In addition to the uptake of nutrients from host cells, parasites also encode transporters to dump waste products. Formate-nitrite transporters (FNT) identified in *Toxoplasma* and *Plasmodium* were thought to mediate lactate efflux in these organisms (18, 19). The single FNT in *Plasmodium* is a potential drug target, given its critical role during parasite growth (20). On the other hand, *Toxoplasma* has three FNTs, all of which are shown to be dispensable for tachyzoite growth *in vitro* (18).

Genomic analyses indicate the presence of a large number of putative transporters in apicomplexan parasites (21). However, their exact substrates and physiological functions are largely unknown. Phosphorous is an essential element for all living organisms and is involved in many biological processes, such as macromolecule synthesis (such as nucleic acids and phospholipids), phosphorylation modification, etc. (22). The import of phosphate from the environment or food is a common way to acquire phosphorous, and this process requires specific transporters. In Arabidopsis thaliana, coordinated actions of multiple phosphate transporters are needed to satisfy the demands for phosphate. The Pht1 family transporters in the root cell membrane mediate phosphate uptake at the root-soil interface (23). To avoid phosphate accumulation in cytosol, phosphate can be further imported to and stored in chloroplasts, mitochondria, and vacuoles through Pht2, Pht3, Pht4, and Pht5 family transporters (24–27). Vacuoles are the primary sites to store excess phosphates (28). The vacuolar phosphate pool can be remobilized to other cellular compartments where it is required. As reported for Oryza sativa, OsVPE1 and OsVPE2 contribute to phosphate efflux from vacuoles (29). Plants mainly obtain phosphorous from the soil in the form of inorganic phosphates, but they can also use organic phosphates, through mutualistic microbes or secretion of phosphatases into the environment to transform organic phosphates into inorganic phosphates (30, 31). Saccharomyces cerevisiae also has multiple inorganic phosphate transporters that work under different conditions (32). In addition, it also has a glycerophosphoinositol transporter, GiT1, that facilitates the uptake of glycerophosphoinositol, which can be catabolized to release the phosphate group and acts as a phosphate source to support yeast growth under phosphate-deficient conditions (33).

Apicomplexan parasites, as obligate intracellular pathogens, obtain phosphates directly from their host cells. PfPiT from Plasmodium falciparum uses the sodium gradient between erythrocyte cytosol and parasite cytosol to drive the uptake of inorganic phosphate into parasites (34). The Na^+^-dependent P_i_ transporting characteristics of PfPiT are well examined, but its physiological importance in parasites has not been established. The *Toxoplasma* ortholog (TgPiT) of PfPiT shows similar phosphate-transporting activity. TgPiT deletion results in reduced inorganic phosphate import and polyphosphate synthesis (35). Unexpectedly, the Δ*Tgpit* mutants only showed a modest growth defect and virulence attenuation (35). Given the essential role of phosphorous to all cells, these findings suggest that there must be other phosphate acquisition pathways in *Toxoplasma*. In this study, we identified a novel protein that had inorganic phosphate transport activity in T. gondii. This new protein, phosphate transporter 2 (TgPT2), is present only in coccidia parasites and is essential for the growth and survival of *Toxoplasma* tachyzoites.

## RESULTS

### Putative phosphate transporters in *Toxoplasma* and related parasites.

**FIG 1:**
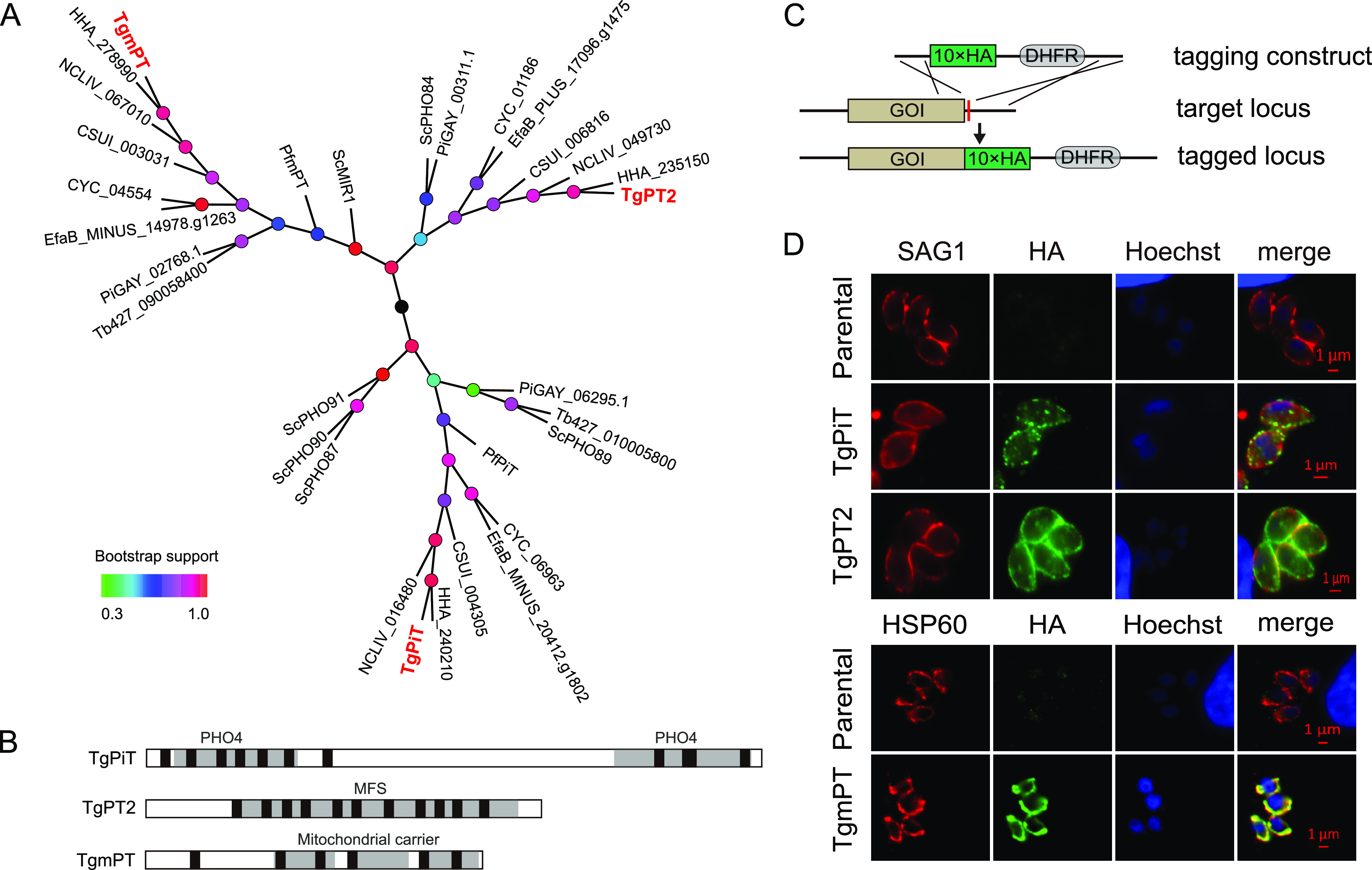
Identification of three putative phosphate transporters in *Toxoplasma*. (A) Phylogenetic analysis of putative phosphate transporters in apicomplexan parasites. Sequence alignments of 31 putative phosphate transporters were performed with ClustalX2, and an unrooted tree was constructed using MEGA 6.06 with the maximum likelihood algorithm. Bootstrap support values ranging from 0.3 to 1.0 are depicted by color gradients. Tg, Toxoplasma gondii; Pf, Plasmodium falciparum; HHA, Hammondia hammondi; NCLIV, Neospora caninum Liverpool strain; EfaB, Eimeria falciformis Bayer Haberkorn; Tb, Trypanosoma brucei; CYC, Cyclospora cayetanensis; CSUI, *Cystoisospora suis*; Sc, Saccharomyces cerevisiae; Pi, Pythium insidiosum. (B) Conserved domains in putative *Toxoplasma* phosphate transporters. Transmembrane regions (TM) are drawn as black boxes. (C) Strategy for C-terminal tagging of gene of interest (GOI) at the endogenous locus. (D) Immunofluorescent staining to determine the subcellular localization of target proteins, which were tagged with HA at the C termini as shown in panel C. SAG1 and HSP60 were included as parasite surface and mitochondrion-specific markers, respectively. Untagged parental strains were included as negative controls.

To identify potential phosphate transporters in *Toxoplasma*, BLAST searches of the *Toxoplasma* genome were performed using known phosphate transporters from S. cerevisiae as baits. Three proteins with significant homology with yeast phosphate transporters were identified. The first one, TGGT1_240210, shows homology to ScPHO89 (Fig. 1A and Fig. S1 in the supplemental material) and contains two PHO4 domains that are found in Na^+^-phosphate symporters (Fig. 1). This protein was named TgPiT, and it has Na^+^-dependent inorganic phosphate transport activity, as reported recently (35). TgPiT is widely present and conserved in apicomplexan parasites, including *Plasmodium* (Fig. 1A), whose PiT was also shown to mediate P_i_ import into parasites in a Na^+^-dependent manner (34). The second one, TGGT1_235150, with homology to PHO84 and GiT1 of yeasts (Fig. S2), encodes a major facilitator superfamily (MFS) protein of 563 amino acids (Fig. 1A and B). This protein has not been described elsewhere and is called TgPT2 here. In contrast to TgPiT, TgPT2 is only found in certain cyst-forming coccidian parasites such as *Eimeria* and *Cystoisospora*, as well as in some fungal species (Fig. 1A). The third one, TGGT1_278990, with high similarity to the mitochondrial phosphate transporter (MIR1) in S. cerevisiae (Fig. S3), is also a novel protein not characterized before and is named *T. gondii* mitochondrial phosphate transporter (TgmPT) here. Multiple sequence analyses suggest that mPT is conserved in most eukaryotic cells (Fig. 1A). When these three putative phosphate transporters and their related proteins in other organisms were included in a phylogenetic analysis, it was found that they formed three separate clades (Fig. 1A), suggesting that they likely have different evolutionary paths and working mechanisms, as well as distinct physiological functions.

### Localization of putative phosphate transporters in *Toxoplasma*.

To examine the expression and subcellular localization of the three putative phosphate transporters identified in T. gondii, a spaghetti-monster HA (smHA) tag was fused to the C terminus of each protein at the endogenous gene locus (Fig. 1C). Indirect immunofluorescent assays (IFA) on the corresponding transgenic parasites demonstrated that TgPiT and TgPT2 were mainly on the parasite surface, as indicated by the colocalization with the plasma membrane marker SAG1. Notably, TgPiT and TgPT2 were also detected in intracellular compartments. This was also reported in a recent study of TgPiT, which was shown to be in the vacuolar compartment and cytoplasmic vesicles in addition to the plasma membrane (35). TgmPT, on the other hand, colocalized with the mitochondrial marker HSP60. Given the presence of six predicted transmembrane motifs, TgmPT is predicted to be on the mitochondrial membrane like its orthologs in other species. During the examination of the immunofluorescent signals, it was noticed that the signal for TgPiT was always lower than that of the other two, although they were tagged with the same epitope at their endogenous loci. This suggests that the expression level of TgPiT was lower than that of TgPT2 and TgmPT, which is consistent with their transcript levels (Fig. S4A and C).

### TgPiT and TgmPT are dispensable for *Toxoplasma* growth and virulence.

**FIG 2:**
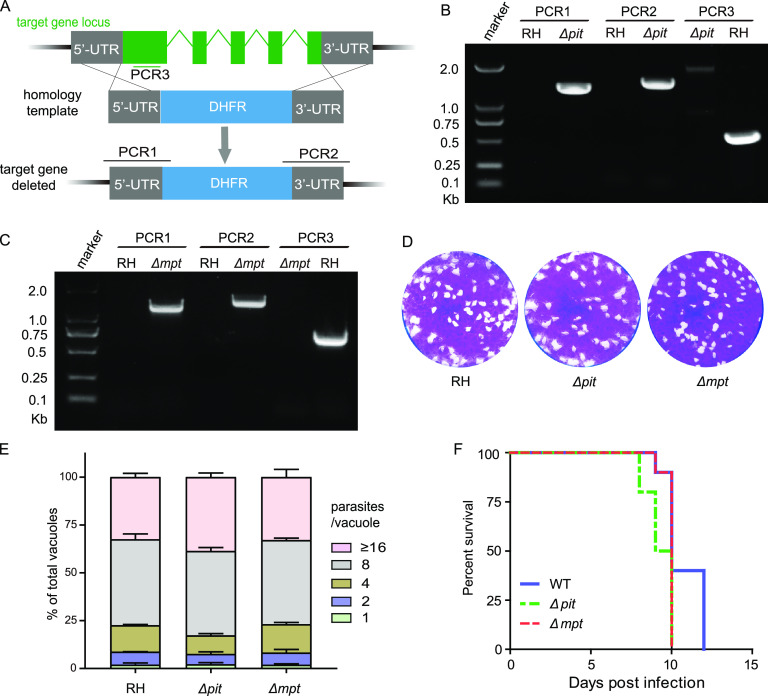
Construction and characterization of Δ*pit* and Δ*mpt* mutants. (A) Diagram of target gene deletion through CRISPR/Cas9-assisted homologous gene replacement. PCR1/2/3 denote diagnostic PCRs for positive clone identification. (B and C) Diagnostic PCRs on a representative Δ*pit* and a Δ*mpt* clone, respectively. (D) Plaque assay assessing the overall growth of the Δ*pit* and Δ*mpt* mutants. (E) Intracellular replication of the Δ*pit* and Δ*mpt* mutants 24 h postinfection of HFF host cells. Means ± SEM (*n* = 3 assays). (F) Virulence test of the Δ*pit* and Δ*mpt* mutant strains in a mouse infection model; *n* = 10 mice/group.

To evaluate the physiological significance of these putative phosphate transporters in *Toxoplasma* parasites, their coding genes were subjected to knockout studies. *TgPiT* and *TgmPT* were successfully deleted in the type 1 strain RH, using CRISPR/Cas9-assisted homologous gene replacements (Fig. 2A). Both were replaced by the selection marker *DHFR*, which was confirmed by diagnostic PCRs (Fig. 2B and C). Deletion of *TgPT2* using the same strategy failed, implying that it might have critical roles for parasite growth or survival.

Plaque assays that determine the overall growth of parasites indicate that deleting *TgPiT* or *TgmPT* did not affect tachyzoite growth *in vitro* (Fig. 2D). Similarly, the efficiencies of intracellular replication of the *TgPiT* or *TgmPT* deletion mutants was indistinguishable from that of the parental strain RH (Fig. 2E). When used to infect mice, the *TgPiT* and *TgmPT* knockouts displayed similar virulence as RH, since all infected mice developed similar symptoms of acute toxoplasmosis and eventually died within 12 days postinfection. Together, these results suggest that the widely present *PiT* and *mPT* are dispensable for *Toxoplasma* growth. On the other hand, the TgPT2, which is only present in cyst-forming coccidia, is likely crucial for parasite survival.

### Conditional depletion of TgPT2 causes growth arrest in *Toxoplasma*.

**FIG 3:**
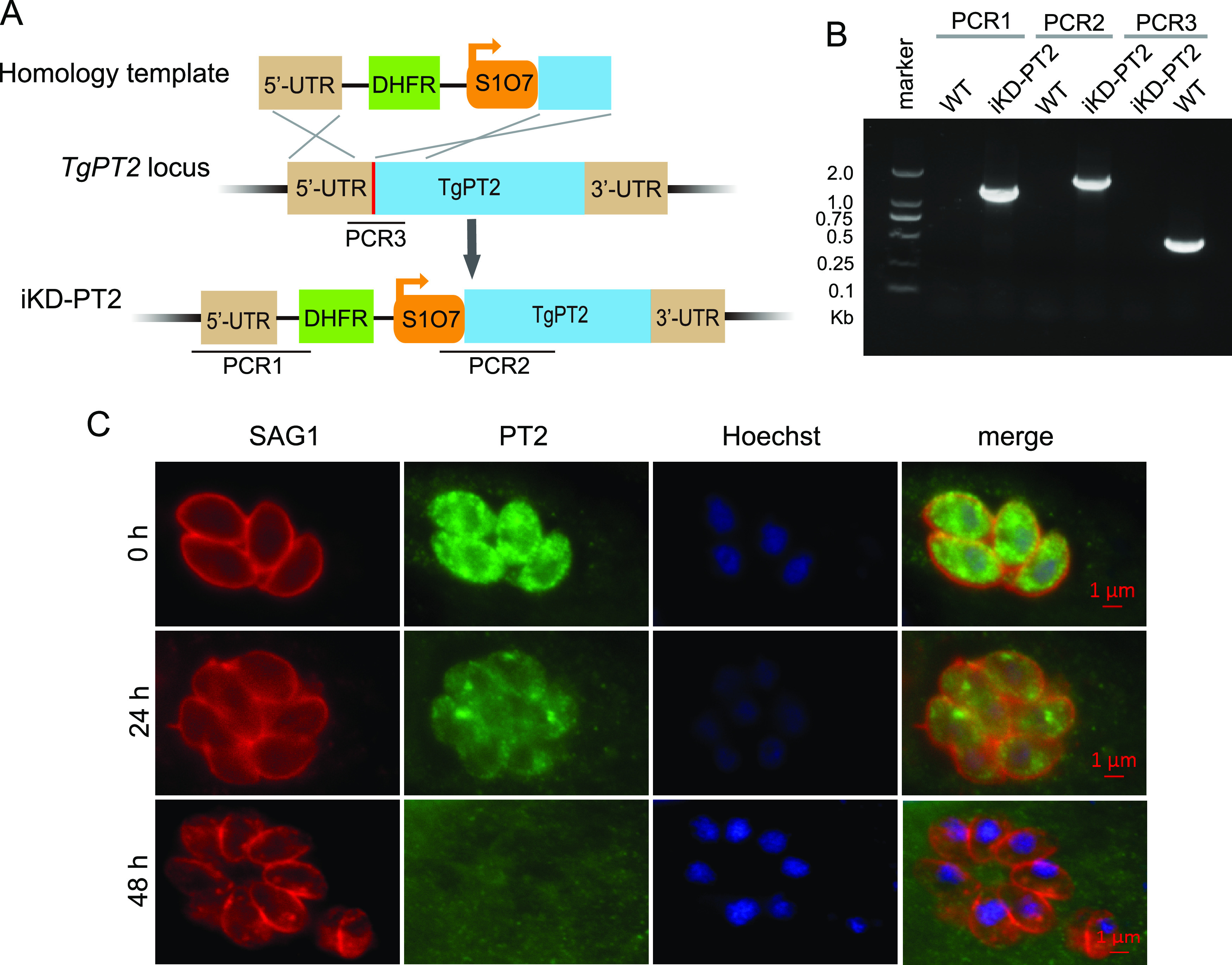
Generation of a conditional depletion strain (iKD-PT2) for TgPT2. (A) Schematic illustration of inserting an S1O7 promoter into the TgPT2 endogenous locus to regulate its expression by ATc. The red bar indicates the CRISPR targeting site, and PCR1/2/3 denote diagnostic PCRs for positive clone identification. (B) Diagnostic PCRs on one iKD-PT2 clone. (C) Immunostaining demonstrating the depletion of TgPT2 expression by 0.5 μg/mL ATc treatment for 0, 24, or 48 h. PT2 was detected using a polyclonal antibody recognizing TgPT2, and SAG1 staining was included as a reference.

**FIG 4:**
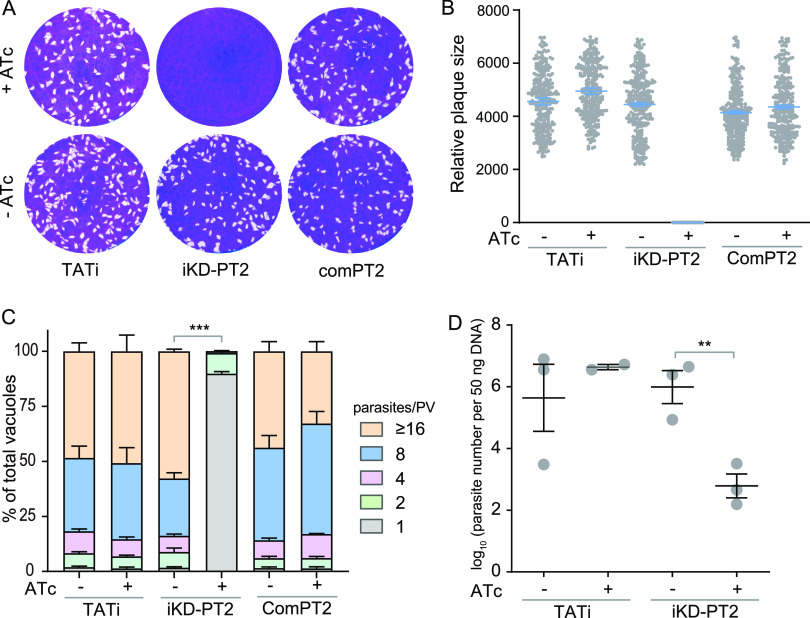
TgPT2 is required for parasite growth both *in vitro* and *in vivo*. (A) Plaque assays comparing the growth of strains in the presence or absence of 0.5 μg/mL ATc. (B) Relative sizes of plaques in panel A. Mean ± SEM of over 200 plaques for each strain from three independent experiments. (C) Intracellular replication of indicated strains with or without ATc treatment. Parasites in the ATc treatment group were pretreated with ATc for 48 h to deplete PT2 expression before assessing their replication rates. Means ± SEM of three biological replicates. ***, *P < *0.001; two-way ANOVA. (D) Parasite burden in peritoneal fluids of IFN-γ^–/–^ mice infected with TATi or iKD-PT2 parasites that were treated with or without 0.2 mg/mL ATc in drinking water. Mean ± SEM; *n* = 3 mice/group. **, *P < *0.01. Unpaired Student’s *t* test.

To study the physiological significance of TgPT2, we generated a conditional knockdown strain (iKD-PT2) whose *PT2* expression could be regulated by anhydrotetracycline (ATc). To this end, the native promoter of *TgPT2* in the TATi strain was replaced by the ATc-regulatable promoter, S1O7 (Fig. 3A). Diagnostic PCRs demonstrated the correct integration of the S1O7 promoter in the iKD-PT2 strain (Fig. 3B). ATc treatment gradually suppressed the expression of *TgPT2* in the iKD-PT2 mutant. The majority of plasma membrane localized PT2 disappeared 24 h after treatment and was reduced to undetectable levels within 48 h (Fig. 3C). Consistent with the key role of PT2, ATc treatment completely blocked plaque formation of the iKD-PT2 mutant in human foreskin fibroblasts (HFF) monolayers (Fig. 4A and B). The plaque assay gives an overall assessment of parasites’ fitness. To further dissect the defects that might cause the growth arrest of PT2-depleted mutants, rates of gliding motility, host cell invasion, egress, microneme secretion, and intracellular replication were determined. Upon ATc treatment, the gliding of tachyzoites on a bovine serum albumin (BSA)-coated surface was decreased by 50%, as estimated by the trail length from SAG1 staining (Fig. S5A and B). Similarly, depletion of PT2 by ATc reduced the rate of host cell invasion by roughly 30% (Fig. S5C). Although PT2 depletion led to reduced parasite motility and invasion efficiency, it did not affect parasites’ egress from HFF cells (Fig. S5D), nor did it affect microneme secretion as determined by the normal release of micronemal protein 2 (MIC2) into the supernatant (Fig. S5E). Strikingly, the PT2-deficient parasites were almost completely unable to proliferate in HFF cells. When PT2-positive parasites were allowed to replicate in HFF cells for 24 h, half of the PVs contained 16 or more parasites. In contrast, the majority (>80%) of the vacuoles of the PT2-depleted mutants contained just one parasite, suggesting that no replication occurred after invasion (Fig. 4C). Together, these results indicate that PT2 is required for efficient parasite motility and host cell invasion and is essential for parasite propagation within host cells.

To further confirm that the observed defects in the TgPT2 depletion mutant are indeed caused by the absence of TgPT2, a complementation strain (comPT2), which had a *PT2* expression cassette inserted into the *UPRT* locus of the iKD-PT2 mutant (Fig. S6A), was constructed. Diagnostic PCRs confirmed the correct integration of the PT2-expressing cassette (Fig. S6B), whereas immunofluorescent staining proved the expression and correct localization of complementing PT2 (Fig. S6C). When the comPT2 strain was used in plaque and replication assays, it was shown that ATc treatment did not affect its plaque formation or intracellular proliferation (Fig. 4A and C). These results suggest that PT2 complementation is able to fully rescue the growth defects of the PT2 depletion mutants.

The strong growth defects of the *PT2* mutant *in vitro* prompted us to check its role *in vivo*. Due to the low virulence of the parental strain TATi, a classic virulence test that monitors the survival of infected mice is challenging. As such, we estimated the parasite propagation *in vivo* by determining the parasite loads in peritoneal fluids of gamma interferon (IFN-γ) knockout mice 9 days after infection. Quantitative PCR results suggest that ATc treatment significantly impaired the propagation of the iKD-PT2 mutant in mice, leading to a 1,000-fold reduction in parasite loads compared to PT2-expressing strains (Fig. 4D). Therefore, PT2 is crucial for parasite growth both *in vitro* and *in vivo*.

### Phosphate transport activities of TgPiT and TgPT2.

**FIG 5:**
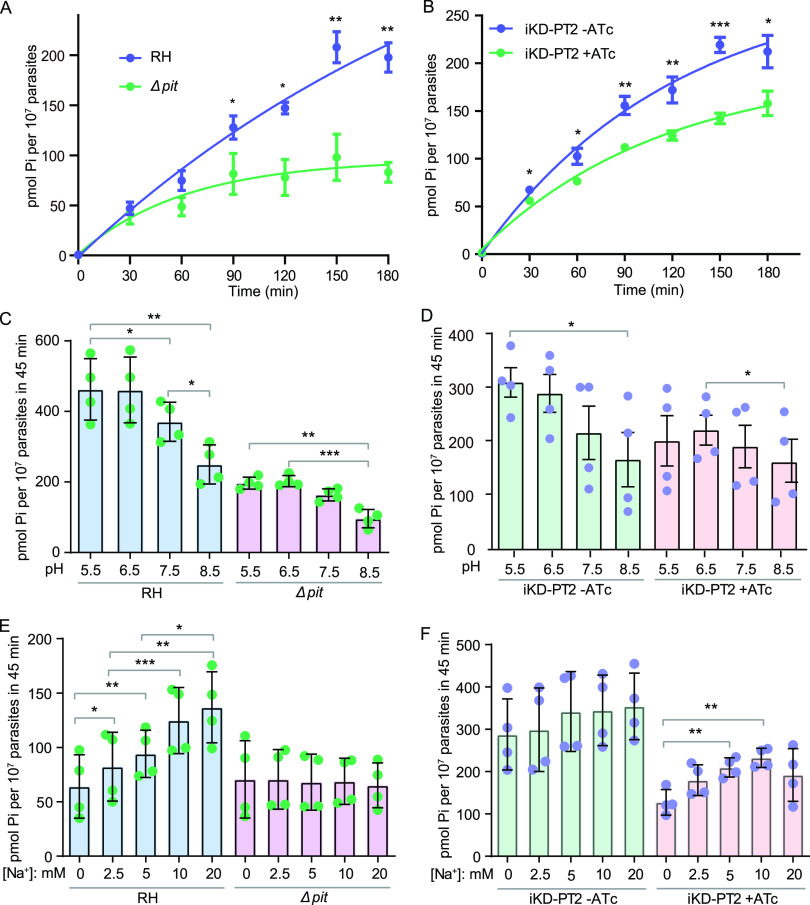
Characterization of the phosphate import activities of the PiT- and PT2-deficient mutants. (A and B) Time-dependent P_i_ uptake of the indicated strains. Parasites were first starved for P_i_ for 12 h at the intracellular growth stage, and then the uptake of ^32^P_i_ over time was measured with purified extracellular tachyzoites. (C and D) pH-dependent P_i_ uptake of the indicated strains. P_i_-starved parasites were allowed to take up ^32^P_i_ for 45 min in media with pH ranging from 5.5 to 8.5. (E and F) Effect of Na^+^ concentration on P_i_ uptake in different strains. The assays were done as in panels C and D, but in the Na^+^-dependent P_i_ transport buffer containing different concentrations of Na^+^. Choline chloride was used to balance the osmolarity of the reaction buffer. In all experiments, iKD-PT2 parasites were pretreated with or without ATc for 48 h before testing. Mean ± SEM; *n* = 4 (*n* = 6 for panel B) independent experiments. *, *P < *0.05; **, *P < *0.01; ***, *P < *0.001. Student’s *t* test.

Both PiT and PT2 were predicted to have phosphate transport activity based on homology analyses, and both were on the plasma membrane of the parasites (Fig. 1). To examine their phosphate transport activity and to determine their contribution to the phosphate uptake of the parasites, an inorganic phosphate transport assay using radioactive ^32^P_i_ was performed. Time-dependent P_i_ uptake assays suggest that import of ^32^P_i_ into purified tachyzoites of the wild-type strain RH was almost linear with time for the first 2 h (Fig. 5A). The P_i_ uptake rate was about 1 pmol/10^7^ parasites/min, similar to what was reported before (35). TgPiT deletion led to reduced P_i_ import into parasites, which was almost saturated within 90 min (Fig. 5A), suggesting that PiT is indeed involved in P_i_ transport in tachyzoites. Time-dependent P_i_ uptake of the iKD-PT2 strain without ATc treatment was similar to that of RH, but the uptake activity was slightly higher (Fig. 5B). This is likely due to the higher expression of PT2 in iKD-PT2 (in which the expression was driven by the S1O7 promoter) than that in RH (expression was driven by the native promoter). ATc treatment significantly reduced the P_i_ transport of the iKD-PT2 parasites (Fig. 5B). It is worth noting that inactivation of neither PiT nor PT2 completely blocked P_i_ import, suggesting that both proteins contributed to parasites’ P_i_ uptake.

The P_i_ transport activity of many phosphate transporters is coupled to cross-membrane transport of Na^+^ or H^+^. To determine the ion dependency of TgPiT and TgPT2, the P_i_ transport assays were performed in buffers with different pH or Na^+^ concentrations (isotonic adjustment with choline chloride). In parasites that expressed both PiT and PT2 (RH and iKD-PT2 without ATc treatment), the P_i_ import is H^+^ dependent, since the import activity was gradually decreased as the pH of the buffer increased from 5.5 to 8.5 (Fig. 5C and D). At pH 8.5, the P_i_ import activity was almost half of that at pH 5.5. Interestingly, both the Δ*PiT* and *PT2* depletion (iKD-PT2, +ATc) mutants showed a similar pH dependency for P_i_ import (Fig. 5C and D), suggesting that both PT2 and PiT are H^+^ dependent. Alternatively, higher P_i_ influx under acidic conditions than basic conditions suggests that H_2_PO_4__−_ may be the preferred substrate for both PiT and PT2, while the transport process through either protein may be pH independent. The preference for H_2_PO_4__−_ was also demonstrated before for PiT (35). When tested over a range of Na^+^ concentrations, the P_i_ import of the RH strain and TATi parasites was strongly Na^+^ dependent (Fig. S7 and Fig. 5E). The Na^+^ dependency completely disappeared in the Δ*PiT* mutant. Moreover, the P_i_ import activity of the Δ*PiT* mutant was comparable to that of the RH strain at 0 mM Na^+^ (Fig. 5E), suggesting that PiT is the main Na^+^-dependent P_i_ transporter in *Toxoplasma*, and PT2 (or additional phosphate transporters, if there are any) is probably Na^+^ independent. Consistent with this, the P_i_ transport in the iKD-PT2 strain without ATc treatment was not obviously Na^+^ dependent, likely because of the higher expression of PT2 in this strain than that in RH. However, after ATc treatment, the P_i_ import of iKD-PT2 was clearly Na^+^ dependent, as PiT became the major phosphate transporter after PT2 depletion (Fig. 5F). Taken together, these results suggest that the P_i_ uptake through TgPiT was Na^+^ dependent, whereas that through PT2 was not. Both proteins had higher P_i_ transport activity under acidic pH, implying that either H_2_PO_4__−_ is the preferred substrate or their transporting activity is H^+^ dependent.

### TgPiT and TgPT2 partially restore the growth of yeast mutants lacking P_i_ transporters.

To further investigate the P_i_ transport functions of TgPiT and TgPT2, they were expressed in the yeast mutant YP100, which expressed *PHO84* under the *GAL1* promoter and contained null mutations for all endogenous P_i_ transporters (Δ*pho84* Δ*pho87* Δ*pho89* Δ*pho90* Δ*pho91* Δ*git1*). This mutant strain grew well with galactose (29). Therefore, when grown on agar plates containing galactose, the growth of YP100 transformed with TgPiT or TgPT2 was similar to that containing the empty vector (Fig. S8). On the other hand, when glucose was supplied as the sole carbon source, the mutant containing the empty vector did not grow or grew very poorly under low P_i_ conditions. Expression of TgPiT and TgPT2 significantly improved the growth under the same conditions, although not as efficiently as PHO84 (Fig. S8). In addition, the capacity of TgPT2 to improve the growth of the yeast mutant seemed similar or even better than that of TgPiT. Together, these data further support that TgPiT and TgPT2 are active phosphate transporters.

### ATP and AMP uptake activity of *Toxoplasma* tachyzoites.

Both TgPiT and TgPT2 displayed inorganic phosphate transport activity in *Toxoplasma*. We also sought to determine whether these transporters were involved in the transport of other compounds. To this end, we added the compounds to be tested to the ^32^P_i_ uptake assays, to see whether they could competitively inhibit the import of P_i_. Nonradioactive P_i_ (^31^P_i_) efficiently inhibited the uptake of ^32^P_i_, which validated the testing system. On the other hand, of the five compounds tested (glucose-6-phosphate, fructose-6-phosphate, fructose-1,6-bisphosphate, myo-inositol, and glycerophosphoinositol), none was able to inhibit the import of ^32^P_i_ (Fig. S9), suggesting that these compounds could not be imported by the P_i_ transport machineries if they were assimilated by the parasites.

**FIG 6:**
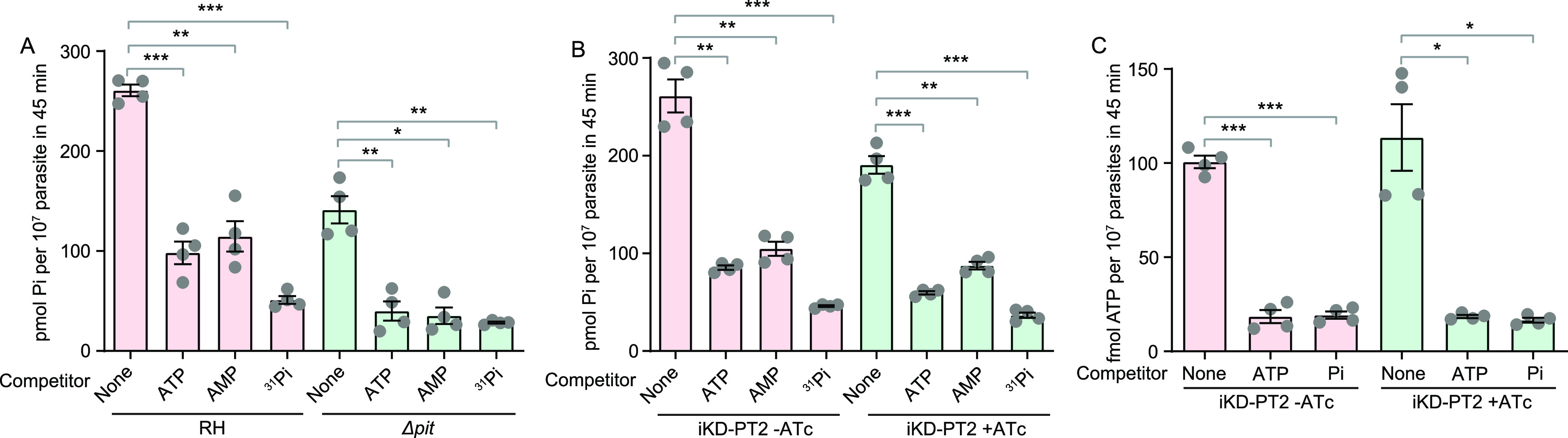
ATP and AMP import activities of *Toxoplasma* parasites. (A and B) Inhibition of ^32^P_i_ transport by ATP and AMP. The ^32^P_i_ uptake assays described in Fig. 5 were performed in the presence of 1 mM ATP, AMP, or KH_2_PO_4_ (^31^P_i_) as competitors. (C) Uptake of ATP by *Toxoplasma* parasites. The iKD-PT2 parasites were pretreated with or without ATc for 48 h and then collected to test their ATP uptake activity using 25 μCi/mL ATP-γ-P^32^. Meanwhile, 1 mM nonradioactive ATP and KH_2_PO_4_ were included in the reaction buffer as competitors. Mean ± SEM; *n* = 4 independent experiments. *, *P < *0.05; **, *P < *0.01; ***, *P < *0.001. Paired two-tailed *t* test.

Using the same competition assays, it was found that both ATP and AMP effectively inhibited the ^32^P_i_ uptake in the RH strain, and the inhibitory efficiencies of ATP and AMP were similar (Fig. 6A). Similar inhibition by ATP and AMP was also observed in the iKD-PT2 strain without ATc treatment (Fig. 6B). To test whether the inhibition of ^32^P_i_ uptake by ATP or AMP was dependent on TgPT2 or TgPiT, competition assays were performed using the Δ*pit* and TgPT2-depleted parasites. Interestingly, although the Δ*pit* and TgPT2-depleted strains had lower ^32^P_i_ uptake activity than their corresponding parental strains, their ^32^P_i_ uptake was also significantly inhibited by ATP or AMP (Fig. 6A and B). Inhibition of ^32^P_i_ uptake by ATP and AMP indicated that the parasites might be able to import these metabolites. To further confirm this, a direct ATP uptake assay was performed using ^32^P-labeled ATP in the iKD-PT2 strain. Radioactive ATP was efficiently absorbed by purified tachyzoites, and such absorption could be effectively inhibited by nonradioactive ATP or P_i_ (Fig. 6C). On the other hand, ATc treatment of the iKD-PT2 strain did not affect the ATP uptake or its inhibition by ATP and P_i_ (Fig. 6C), indicating that the ATP-importing activity is independent of PT2. To further examine the ATP import activity in *Toxoplasma* parasites, radioactive ATP-α-P^32^ was used to rule out the possibility of hydrolysis of ATP-γ-P^32^ that generated inorganic P^32^ for parasite uptake. The results revealed that ATP-α-P^32^ could also be imported into *Toxoplasma* parasites, and the import was significantly inhibited by P_i_, nonradioactive ATP, and the ATP analog, ATP-γ-S, but not by FBP (Fig. S10). Taken together, these results suggest that *Toxoplasma* tachyzoites are able to take up ATP and AMP from the environment, but PT2 and PiT are not the main transporters responsible for their uptake.

### Inactivation of phosphate transporters alters gene expression in T. gondii.

**FIG 7:**
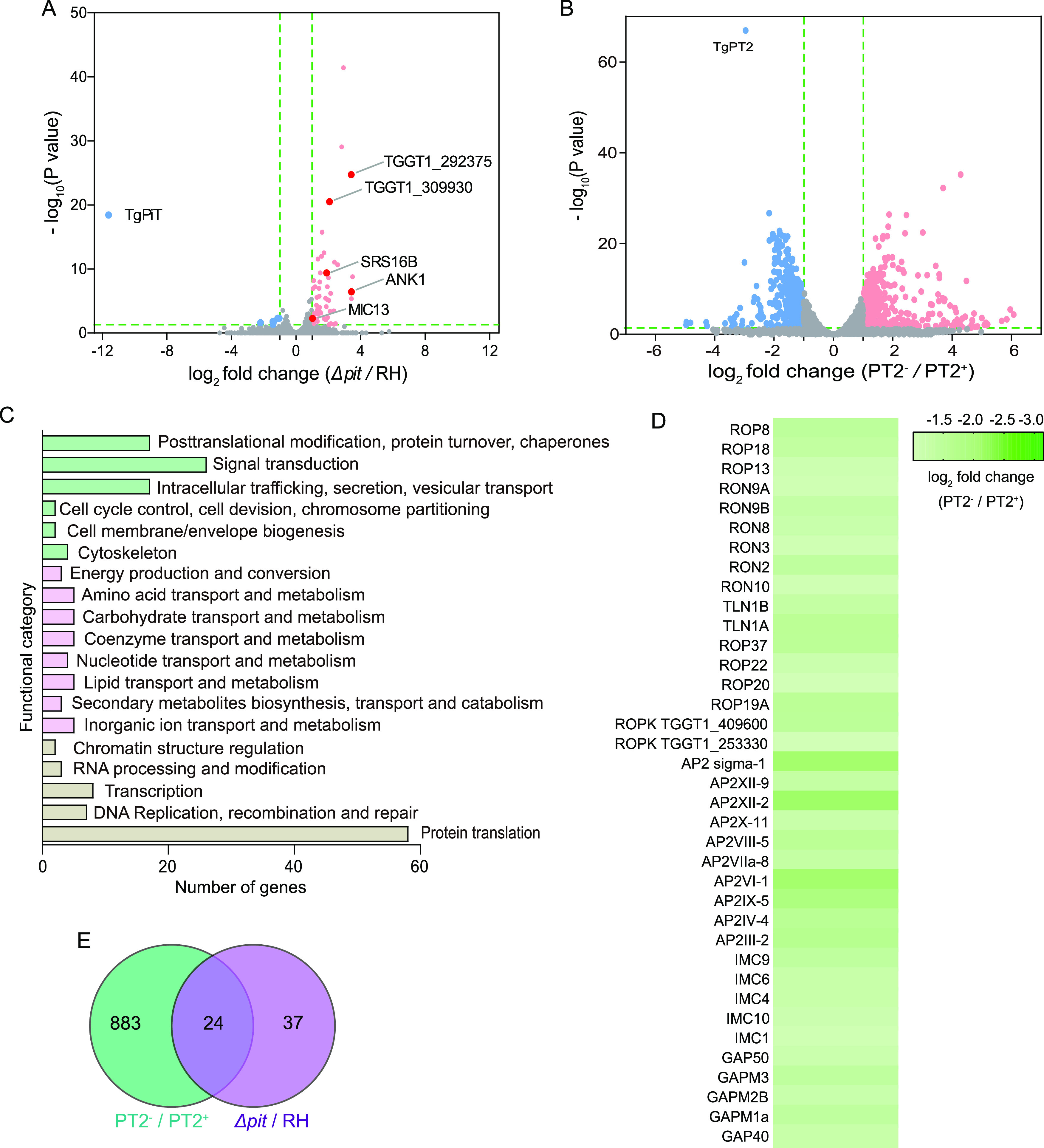
Transcriptomic analyses of the *PiT*- and *PT2*-defective mutants. (A and B) Volcano plot showing the differentially expressed genes in Δ*pit* compared to the parental strain RH and PT2-/PT2^+^, respectively. The blue and red dots represent down- and upregulated genes, respectively. The dark red dots with gene names displayed indicate the genes that are typically upregulated during the conversion of tachyzoites to bradyzoites. (C) COG analysis of differentially expressed genes before and after *TgPT2* knockdown. (D) Heatmap showing the decreased expression of selected AP2 factors, rhoptry proteins, IMC, and glideosome-associated proteins upon *TgPT2* suppression. Data were extracted from the RNA-seq analyses, and the mean expression changes of each gene from three independent samples were plotted. (E) Venn diagram showing the similarity of gene expression changes after *PiT* and *PT2* disruption.

To estimate how *Toxoplasma* parasites respond and adapt to decreased phosphate uptake due to PiT or PT2 inactivation, RNA sequencing (RNA-seq) analyses were performed to assess their gene expression changes. Compared with the parental strain RH, the abundance of 61 genes was significantly changed in the Δ*Tgpit* mutant (Fig. 7A). The modest gene expression change associated with *PiT* disruption is consistent with the lack of an obvious growth phenotype of this mutant. Of the 61 differentially expressed genes, the vast majority were upregulated in the Δ*Tgpit* mutant (Table S3), among which, TGGT1_226100, encoding a haloacid dehalogenase (HAD) family hydrolase, was increased 3.1-fold in the Δ*Tgpit* mutant. Since many HAD-containing proteins have phosphohydrolase activity, this may suggest that increasing phosphomonoester hydrolysis is a strategy for the parasites to adapt to TgPiT deficiency. *TgPiT* inactivation also led to increased expression of several genes whose products mediate protein-protein interactions. These include TGGT1_266860 (BTB/POZ domain-containing protein, increased 11.3-fold), TGGT1_216140 (tetratricopeptide repeat-containing protein ANK1, increased 10.9-fold) and TGGT1_202280 (G-beta repeat-containing protein, increased 3.6-fold). Loss of *TgPiT* also resulted in elevated expression of enzymes that are involved in signaling molecule production, such as TGGT1_257945 (increased 4.1-fold), which encodes a 3′5′-cyclic nucleotide phosphodiesterase domain-containing protein. These proteins might alter the signaling pathways in the parasites in response to *PiT* deletion. In addition, one transporter belonging to the major facilitator family (TGGT1_293420, 2.2-fold change) with unknown substrates was upregulated due to loss of TgPiT. Several genes (ANK1, MIC13, bradyzoite surface antigen SRS16B, TGGT1_292375, and TGGT1_309930), whose expressions are typically increased during transition from tachyzoites to bradyzoites (Fig. 7A), were upregulated in the Δ*Tgpit* mutant. This suggests that TgPiT deletion and the resulting decreased P_i_ uptake might cause a stress response in the parasite.

In contrast to *TgPiT* disruption, loss of *TgPT2* caused a more dramatic change in gene expression, with 455 genes being upregulated and 452 genes downregulated (Fig. 7B and Table S4). Functional enrichments of these differentially expressed genes (DEGs) using the cluster of orthologous groups (COG) indicate that these DEGs were enriched in pathways such as genetic information processing (such as protein translation, transcription, RNA processing and modification, DNA replication and repair, and chromatin structure regulation), nutrient transport and metabolism, signal transduction, intracellular trafficking, protein secretion, vesicular transport, etc. (Fig. 7C). Among these DEGs, a subset of AP2 factors and rhoptry proteins (ROPs and RONs) were found to be significantly downregulated upon *PT2* depletion (Fig. 7D). Many of these genes have low phenotype scores in a genome-wide screen, indicating their critical roles in optimal parasite growth (36). RON proteins are shown to be essential for parasite invasion into host cells (37, 38). Therefore, reduced expression of these genes might contribute to the poor growth of the *PT2* depletion mutant. In addition, a number of inner membrane complex (IMC) proteins involved in IMC biogenesis were also significantly downregulated (Fig. 7D), which might also affect parasite growth due to the crucial role of IMC in parasites.

When the DEGs resulting from *TgPiT* deletion and those from *TgPT2* depletion were compared, it was found that a significant number of them were shared. Of the 61 DEGs found in the Δ*Tgpit* mutant, 24 were also found to be differentially expressed upon *TgPT2* depletion (Fig. 7E). These include the above-mentioned major facilitator transporter (TGGT1_293420), BTB/POZ domain-containing protein (TGGT1_266860), G-beta repeat-containing protein (TGGT1_202280), and ANK1 (TGGT1_216140), as well as a vitamin K epoxide reductase family protein (TGGT1_203720) that might be involved in vitamin K cycle and calcium homeostasis (Fig. 7E). The similar gene expression changes caused by inactivation of *TgPiT* and *TgPT2* suggest that the two proteins have overlapping functions.

## DISCUSSION

The apicomplexan phylum contains a variety of pathogenic protozoa that pose serious threats to the health of humans and animals. A common feature of these organisms is that they are all obligate intracellular parasites, and they take up all necessary nutrients from host cells to support their growth and development. Yet the underlying mechanisms of nutrient salvage are largely unexplored. Phosphorous is an essential element for all living cells, and it is imported into cells in the form of inorganic or organic phosphates (23, 32, 33). In this study, we identified three putative phosphate transporters in *Toxoplasma*, two of which, TgPiT and TgPT2, were localized in the plasma membrane and involved in phosphate uptake from the environment. The third one, TgmPT, was localized to the mitochondrion and is probably a mitochondrial phosphate transporter, given the high sequence similarity with known mPTs (25). Inactivation of each of these genes suggests that deletion of TgPiT or TgmPT is well tolerated, whereas TgPT2 is essential for parasite growth. Moreover, TgPT2 orthologs are restricted to cyst-forming coccidia parasites such as *Toxoplasma*, *Eimeria* and *Cystoisospora* and are absent in the mammalian hosts. Therefore, it is a feasible target for drug design.

TgPT2 and TgPiT are both localized to the plasma membrane, as well as intracellular compartments, which are likely the plant-like vacuole and/or cytoplasmic vesicles, as recently reported for TgPiT (35). They also both have P_i_ transport activity. Then the question becomes why PT2 is essential for tachyzoite growth whereas PiT is completely dispensable. The dispensability of PiT may be explained by the P_i_ transport activity function of PT2. Consistent with this, the Δ*pit* mutant is still able to take up P_i_ from the medium. In the P_i_ import experiment, the P_i_ uptake kinetics of the Δ*pit* mutant is similar to that of the wild-type strain RH in the first 30 min (Fig. 5A). In contrast, the *PT2* depletion mutant displayed reduced P_i_ uptake at all time points (Fig. 5B). When the time of uptake was extended, the Δ*pit* mutant exhibited a more pronounced reduction in P_i_ import than the *PT2* depletion mutant. This implies that PT2 is mainly responsible for P_i_ uptake when the P_i_ level in parasites is low, whereas PiT is more important to boost the P_i_ level when cellular P_i_ reaches a certain level. In addition to the different working modes of PiT and PT2, it is also possible that they have different substrate specificities. PT2 may transport other nutrients besides inorganic phosphate. Using a competition assay, we have examined the tachyzoites’ ability to transport phosphate-containing organic compounds such as glucose-6-phosphate and fructose-6-phosphate. However, none of the compounds tested was able to inhibit parasites’ P_i_ uptake (Fig. S9). Sequence analyses suggest that PT2 has limited homology to the glycerophosphoinositol (GPI) transporter in S. cerevisiae (39). Nonetheless, competition assays showed that GPI was not able to compete with P_i_ import (Fig. S9), suggesting that PT2 probably does not transport GPI either. Therefore, the additional substrates of PT2, if any, remain to be determined. Lastly, all the parasite transport assays were performed with extracellular parasites. While T. gondii is an obligate intracellular parasite, all the nutrients supporting its growth are directly derived from the host cells. Therefore, the use of intracellular parasites for the P_i_ uptake assays would be the best to illustrate the role of each of these transporters. Nonetheless, this is technically challenging. Due to the differences between the intracellular environments and the buffer conditions used here for the transport assays, we should bear in mind that the properties of the transporters deduced from extracellular parasite-based experiments may not exactly reflect what is occurring during the intracellular stage. Consistent with this notion, when used to complement a yeast mutant that is defective in P_i_ transport, both TgPiT and TgPT2 could improve the yeast growth under low-P_i_ conditions. In addition, their capacity of improving the growth of yeast seemed indistinguishable, suggesting similar activities in this case.

When ATP and AMP were used as competitors in the P_i_ import assay, surprisingly, it was found that both ATP and AMP efficiently inhibited the P_i_ uptake in the wild-type, Δ*pit*, and *PT2* depletion strains (Fig. 6). These results suggest that tachyzoites use P_i_ import-related mechanisms to take up ATP and AMP from the environments. The ATP uptake activity was further confirmed using radioactive ^32^P-ATP (Fig. 6C). The competitive inhibition of ATP import by P_i_ also demonstrates that the mechanism of ATP import is related to P_i_ transport. On the other hand, ATP and AMP do not seem to be imported by PiT or PT2, because ATP and AMP were both able to inhibit P_i_ uptake in the Δ*pit* and *PT2* depletion mutants. In addition, the ^32^P-ATP import capacity of iKD-PT2 was not affected by ATc treatment and was inhibited by P_i_ to similar levels in the presence or absence of PT2 (Fig. 6C). These results imply that there are additional P_i_ transport mechanisms across the parasite plasma membrane and that some of those are involved in ATP and AMP import. T. gondii lacks the *de novo* synthesis pathway for purine nucleotides and relies on hosts for the supply of purines. Early studies showed that extracellular tachyzoites could incorporate adenosine nucleotides into the parasites and that AMP was the preferred substrate (40). Later, it was shown that the parasites mainly take up adenosine nucleosides from host cells or environments and are then converted to AMP by an adenosine kinase (41). While the direct salvage of host ATP and AMP is still being debated, our results, together with the early observations, support the model that tachyzoites are able to take up host ATP/AMP. Interestingly, similar models were also proposed recently for *Cryptosporidium* (42).

Of the two putative plasma membrane localized phosphate transporters we identified in *Toxoplasma*, PiT homologs are widely present in apicomplexan parasites, whereas PT2 is restricted to cyst-forming coccidia. *Plasmodium* parasites express PiT but not PT2, and the Na^+^-dependent P_i_ transport activity of PiT was well demonstrated (34). Nonetheless, its physiological significance in malaria parasites has not been determined. The results from a genome-wide transposon mutagenesis screen suggest that inactivation of PiT in *Plasmodium* would probably reduce the fitness of the parasites (43). This is not surprising when PiT is the only recognizable inorganic phosphate transporter in the plasma membrane of this organism. Interestingly, no homologs of TgPiT or TgPT2 were found in *Crystosporidium* species, implying that they use different mechanisms for phosphate acquisition. Genomic analyses suggest that Cryptosporidium parvum uses a single salvage pathway to provide purine nucleotides (42). However, a number of enzymes in the salvage pathway could be ablated without affecting the viability of the parasites. On the other hand, these mutants are hypersensitive to mycophenolic acid, which inhibits purine nucleotide synthesis in host cells (42). Together, these results imply that C. parvum parasites may be able to import purine nucleotides from the host cells they parasitize. As our work indicated a possible P_i_ transport-related mechanism to import ATP/AMP in *Toxoplasma*, similar mechanisms may exist in C. parvum to import P_i_ and purine nucleotides. Nonetheless, in both parasites, the molecular nature of the ATP/AMP import mechanisms is not yet known and deserves further investigation.

## MATERIALS AND METHODS

### Biological reagents and resources.

All T. gondii strains and their derivative mutants generated in this study were cultured in a confluent monolayer of human foreskin fibroblasts (HFF) as described previously (44). The TgSAG1, TgMIC2, and TgALD antibodies were kindly provided by David Sibley (Washington University, St. Louis, MO, USA). Rabbit anti-SAG2 was a gift from Honglin Jia (Harbin Veterinary Research Institute, People’s Republic of China). Mouse anti-TgHSP60 was produced in our laboratory. Mouse anti-hemagglutinin (HA) was purchased from MBL (Medical & Biological Laboratories, Japan). The fluorophore-conjugated secondary antibodies (Alexa488 or Alexa594) were purchased from Invitrogen (Carlsbad, CA, USA). KH_2_^32^PO_4_ (NEX060001MC), ATP-γ-^32^P, and ATP-α-^32^P (NEG502A001MC and NEG003H) were obtained from PerkinElmer (PerkinElmer, Waltham, MA, USA).

### Phylogenetic analysis.

To identify potential phosphate transporters in T. gondii, BLAST searches were performed in ToxoDB using Saccharomyces cerevisiae inorganic phosphate transporters (PHO84, PHO87, PHO89, PHO90, PHO91), mitochondrial phosphate transporter MIR1, and organic phosphate transporter GiT1 as baits. Hits with E values below e^−20^ were subject to reciprocal BLAST search against the S. cerevisiae protein database. The identified *Toxoplasma* transporters were also used in BLAST searches to identify homologs in other organisms.

For phylogenetic analyses, protein sequences were aligned in ClustalX2. Gaps or poorly conserved regions were trimmed, and the curated sequences were then used to construct phylogenetic trees using MEGA 6.06 with the maximum likelihood algorithm (45). Finally, the tree was annotated and viewed using FigTree 1.4.3 (http://tree.bio.ed.ac.uk/software/Figtree/).

### Construction of plasmids and parasite strains.

All primers and plasmids used in this study are listed in Tables S1 and S2. Locus-specific CRISPR plasmids were generated by replacing the guide RNA (gRNA) sequence of pSAG1::Cas9-U6::sgUPRT with gene-specific gRNAs, through Q5 site-directed mutagenesis (New England Biolabs, USA) as previously described (46).

To endogenously tag genes with spaghetti-monster HA (smHA) tag at the C termini, a donor sequence containing the smHA tag and *DHFR* selection marker was amplified from the *pSL24m-Linker-smFP-DHFR-LoxP-T7* plasmid (47). The resulting amplicon contained two 50-bp homologous arms at the 5′ and 3′ ends, respectively, corresponding to sequences flanking the stop codon of target genes. Subsequently, the donor amplicon and a locus-specific plasmid targeting the 3′ untranslated region (UTR) next to the stop codon were cotransfected into purified RH Δ*ku80* tachyzoites (48). Transfectants were selected with 1 μM pyrimethamine and then examined by immunofluorescent staining to analyze the subcellular localization of target proteins.

Direct knockouts of *TgPiT* and *TgmPT* were constructed by CRISPR/Cas9 directed homologous gene replacement, as described before (44). Briefly, homology templates containing the selection marker DHFR* and the gene-specific CRISPR plasmid were cotransfected into the purified tachyzoites of the RH Δ*hxgprt* strain. Transfectants were selected with 1 μM pyrimethamine, single cloned by limiting dilution, and examined by diagnostic PCRs. To generate the iKD-PT2 conditional knockdown strain, the tetracycline-regulatable promoter S1O7 was used to replace the native promoter of *TgPT2* via double homologous recombination (49). For this purpose, the fragment containing the S1O7 promoter and the selection marker DHFR were cointroduced into the TATi line along with the target-specific CRISPR plasmid. Positive clones were identified by pyrimethamine selection and diagnostic PCRs. Anhydrotetracycline (ATc; TaKaRa, Japan) at a final concentration of 0.5 μg/mL was added into culture medium to suppress the expression of *TgPT2*. Unless otherwise indicated, the iKD-PT2 parasites were treated with or without ATc for 48 h prior to experiments.

To complement the iKD-PT2 mutant, the coding sequence of TgPT2 was amplified from cDNA of the TATi line and cloned into a vector that allowed the expression of PT2-HA (driven by the tubulin promoter) from the *UPRT* locus. The resulting construct was electroporated into iKD-PT2, along with a *UPRT*-targeting CRISPR plasmid. Transfectants were selected with 30 μM chloramphenicol and 10 μM fluorodeoxyuridine (FUDR). Finally, the complementing clones were verified by diagnostic PCRs and immunofluorescent staining.

### TgPT2 antibody production.

The peptide CRKFRRGSRAFE, corresponding to amino acids 552 to 563 of TgPT2, was synthesized and conjugated to keyhole limpet hemocyanin (KLH). Antisera against this conjugated peptide were raised by immunization of two New Zealand White rabbits. The antibodies were affinity-purified and dialyzed in phosphate-buffered saline (PBS) before use.

### Characterization of parasite fitness *in vitro*.

Plaque assays that estimate the overall growth, motility assays that examine the gliding motility of purified tachyzoites on coated surfaces, invasion assays that determine the efficiencies of host cell invasion, Ca^2+^-induced egress assays that assess the egress of parasites from HFF host cells, and microneme secretion assays that check the release of microneme proteins to culture supernatants were performed as previously described (44, 50).

### Transport assays.

Before harvest, intracellular *Toxoplasma* tachyzoites were starved for P_i_ by replacing the culture medium with P_i_-free medium and were cultured for another 12 h. Then parasites were purified and suspended in P_i_-free Dulbecco Modified Eagle medium (DMEM). Unless otherwise indicated, the P_i_ uptake assays in *Toxoplasma* parasites were performed in P_i_-free DMEM (Thermo Fisher Scientific, USA), which contained 110.3 mM sodium chloride and 44.0 mM sodium bicarbonate. Phosphate uptake assays were initiated by adding radioactive KH_2_^32^PO_4_ (final concentration, 2 μCi/mL, 1 nM/μCi) along with 50 μM KH_2_PO_4_ to 200 μL reaction mixture with 1 × 10^7^ tachyzoites. Reaction mixtures were incubated 37°C for different amounts of time and then stopped by adding an equal volume of ice-cold DMEM. Parasites were pelleted and washed 3 times with ice-cold DMEM, and then the radioactivity was quantified with a liquid scintillation counter (Perkin-Elmer, Waltham, MA, USA). The time-dependent radioactive phosphate uptake data were fitted with a single exponential algorithm. The ATP transport assays were done in a similar way, except that final concentrations of 25 μCi/mL (3,000 Ci/mmol) for ATP-γ-^32^P and 4 μCi/mL (800 Ci/mmol) ATP-α-^32^P were used.

To determine the effects of pH on phosphate uptake, the pH of the above-mentioned system was adjusted by HCl or KOH. The Na^+^-dependent transport assay was performed in the Na^+^-dependent P_i_ transport buffer containing 1.5 mM CaCl_2_, 5 mM KCl, 10 mM HEPES (pH 7.4), 1 mM MgCl_2_, 50 μM KH_2_PO_4_, and various concentrations of NaCl (0 to ~20 mM). Choline chloride was used to maintain the osmolarity at about 300 mosM when varying the Na^+^ concentration, as described previously (35). All assays were repeated three or more times independently. The radioactivity of negative controls without parasites was used as a baseline and subtracted from each experimental sample before data analysis.

### Assessing the phosphate transport functions of *Toxoplasma* proteins in yeasts.

The coding sequences of TgPiT, TgPT2, and ScPHO84 were cloned into the yeast expression vector pJR3455A (provided by Jared Rutter, University of Utah School of Medicine) using the ClonExpress II one-step cloning kit (Vazyme Biotech, Nanjing, People’s Republic of China). The resulting recombinant plasmids and the empty vector were individually transformed into the yeast mutant strain YP100 (Δ*pho84* Δ*pho87* Δ*pho89* Δ*pho90* Δ*pho91* Δ*git1* pGal::pho84) (provided by Chuang Wang, Huazhong Agricultural University). The transformed yeast cells were grown on synthetic dropout (-Ura) agar plates with normal P_i_ concentration (7.3 mM) and 2% galactose for 3 to 4 days. Positive transformants were grown in the same medium to an optical density at 600 nm (OD_600_) of 1.0. Cells were collected and washed three times with sterilized water and resuspended to an OD_600_ of 1.0. Serial dilutions (5-fold) of the cultures were spotted on P_i_-free yeast nitrogen bast (YNB) agar plates containing 2% glucose and different concentrations of KH_2_PO_4_. Plates were incubated at 30°C for 5 days, and the colony formation was recorded.

### Animal experiments.

To determine the impact of *TgPiT* and *TgmPT* deletion on parasite virulence *in vivo*, tachyzoites of the RH Δ*hxgprt*, RH Δ*pit*, and RH Δ*mpt* strains were used to infect 7-week-old ICR mice (100 parasites in 200 μL PBS per mouse, 10 mice per strain) through peritoneal injection. Then the symptoms and survival of the mice were monitored daily.

To estimate the effect of TgPT2 depletion on parasite proliferation *in vivo*, 1,000 tachyzoites (in 200 μL PBS) of the TATi or iKD-PT2 strains were used to infect IFN-γ^−/−^ mice (7 weeks old) through peritoneal injection. Then the mice were provided with sterile drinking water with or without 0.2 mg/mL ATc (51). Nine days postinfection, all mice were euthanized, and peritoneal fluids were collected for genomic DNA extraction. Then 50 ng DNA of each sample was used in quantitative PCR analysis to determine the parasite load. A standard curve was produced using serial dilutions of genomic DNA extracted from 5 × 10^6^ TATi tachyzoites.

All animal experiments were conducted in accordance with the National Research Council’s Guide for the Care and Use of Laboratory Animals and were approved by the Huazhong Agricultural University Ethics Committee (approval number HZAUMO-2019-032).

### Transcriptomic analysis.

Freshly egressed tachyzoites of the RH Δ*hxgprt*, RH Δ*pit*, and RH Δ*mpt* strains or the iKD-PT2 mutant with or without 48 h of ATc pretreatment were harvested and purified by 3- μm-membrane filtration and washed twice with prechilled PBS. Then the total RNA of each sample was extracted using the TRIzol reagents following the manufacturer’s instructions (Invitrogen, USA). Three biological replicates were prepared for each strain. Subsequently, the RNA samples were treated with DNase I (TaKaRa, Japan) and then used for library construction using the TruSeq RNA preparation kit (Illumina, San Diego, CA, USA). Paired-end sequencing was done using the Illumina HiSeq X Ten sequencer. High-quality clean reads were mapped to the *Toxoplasma* reference genome (GT1 strain), and differential gene expression was identified using DESeq2 (52).

### Statistics and reproducibility.

All data shown in graphs are presented as the mean ± standard error of the mean (SEM) from three or more independent assays, unless specified otherwise. Statistical analyses were performed using GraphPad Prism software (v8) using two-tailed Student’s *t* test, and one-way or two-way analysis of variance (ANOVA) as specified in the figure legends.

### Data availability.

RNA-seq reads have been deposited to the Gene Expression Omnibus on NCBI (accession number GSE189677).

## References

[1] Seeber F, Steinfelder S. 2016. Recent advances in understanding apicomplexan parasites. F1000Res 5:1369. doi:10.12688/f1000research.7924.1. PMC4909106 27347391

[2] Swapna LS, Parkinson J. 2017. Genomics of apicomplexan parasites. Crit Rev Biochem Mol Biol 52:254–273. doi:10.1080/10409238.2017.1290043. 28276701 PMC6813780

[3] Torgerson PR, Mastroiacovo P. 2013. The global burden of congenital toxoplasmosis: a systematic review. Bull World Health Organ 91:501–508. doi:10.2471/BLT.12.111732. 23825877 PMC3699792

[4] Boothroyd JC, Grigg ME. 2002. Population biology of *Toxoplasma gondii* and its relevance to human infection: do different strains cause different disease? Curr Opin Microbiol 5:438–442. doi:10.1016/s1369-5274(02)00349-1. 12160866

[5] Mendez OA, Koshy AA. 2017. *Toxoplasma gondii*: entry, association, and physiological influence on the central nervous system. PLoS Pathog 13:e1006351. doi:10.1371/journal.ppat.1006351. 28727854 PMC5519211

[6] Vargas-Villavicencio JA, Besne-Merida A, Correa D. 2016. Vertical transmission and fetal damage in animal models of congenital toxoplasmosis: a systematic review. Vet Parasitol 223:195–204. doi:10.1016/j.vetpar.2016.04.024. 27198800

[7] Havelaar AH, Kemmeren JM, Kortbeek LM. 2007. Disease burden of congenital toxoplasmosis. Clin Infect Dis 44:1467–1474. doi:10.1086/517511. 17479945

[8] Montazeri M, Sharif M, Sarvi S, Mehrzadi S, Ahmadpour E, Daryani A. 2017. A systematic review of *in vitro* and *in vivo* activities of anti-*Toxoplasma* drugs and compounds (2006–2016). Front Microbiol 8:25. doi:10.3389/fmicb.2017.00025. 28163699 PMC5247447

[9] Mordue DG, Desai N, Dustin M, Sibley LD. 1999. Invasion by *Toxoplasma gondii* establishes a moving junction that selectively excludes host cell plasma membrane proteins on the basis of their membrane anchoring. J Exp Med 190:1783–1792. doi:10.1084/jem.190.12.1783. 10601353 PMC2195726

[10] Schwab JC, Beckers CJ, Joiner KA. 1994. The parasitophorous vacuole membrane surrounding intracellular *Toxoplasma gondii* functions as a molecular sieve. Proc Natl Acad Sci USA 91:509–513. doi:10.1073/pnas.91.2.509. 8290555 PMC42978

[11] Gold DA, Kaplan AD, Lis A, Bett GC, Rosowski EE, Cirelli KM, Bougdour A, Sidik SM, Beck JR, Lourido S, Egea PF, Bradley PJ, Hakimi MA, Rasmusson RL, Saeij JP. 2015. The *Toxoplasma* dense granule proteins GRA17 and GRA23 mediate the movement of small molecules between the host and the parasitophorous vacuole. Cell Host Microbe 17:642–652. doi:10.1016/j.chom.2015.04.003. 25974303 PMC4435723

[12] Parker KER, Fairweather SJ, Rajendran E, Blume M, McConville MJ, Broer S, Kirk K, van Dooren GG. 2019. The tyrosine transporter of *Toxoplasma gondii* is a member of the newly defined apicomplexan amino acid transporter (ApiAT) family. PLoS Pathog 15:e1007577. doi:10.1371/journal.ppat.1007577. 30742695 PMC6386423

[13] Rajendran E, Hapuarachchi SV, Miller CM, Fairweather SJ, Cai Y, Smith NC, Cockburn IA, Broer S, Kirk K, van Dooren GG. 2017. Cationic amino acid transporters play key roles in the survival and transmission of apicomplexan parasites. Nat Commun 8:14455. doi:10.1038/ncomms14455. 28205520 PMC5316894

[14] Fairweather SJ, Rajendran E, Blume M, Javed K, Steinhofel B, McConville MJ, Kirk K, Broer S, van Dooren GG. 2021. Coordinated action of multiple transporters in the acquisition of essential cationic amino acids by the intracellular parasite *Toxoplasma gondii*. PLoS Pathog 17:e1009835. doi:10.1371/journal.ppat.1009835. 34432856 PMC8423306

[15] Blume M, Rodriguez-Contreras D, Landfear S, Fleige T, Soldati-Favre D, Lucius R, Gupta N. 2009. Host-derived glucose and its transporter in the obligate intracellular pathogen *Toxoplasma gondii* are dispensable by glutaminolysis. Proc Natl Acad Sci USA 106:12998–13003. doi:10.1073/pnas.0903831106. 19617561 PMC2722337

[16] De Koning HP, Al-Salabi MI, Cohen AM, Coombs GH, Wastling JM. 2003. Identification and characterisation of high affinity nucleoside and nucleobase transporters in *Toxoplasma gondii*. Int J Parasitol 33:821–831. doi:10.1016/s0020-7519(03)00091-2. 12865082

[17] Marquez-Nogueras KM, Hortua Triana MA, Chasen NM, Kuo IY, Moreno SN. 2021. Calcium signaling through a transient receptor channel is important for *Toxoplasma gondii* growth. Elife 10:e63417. doi:10.7554/eLife.63417. 34106044 PMC8216714

[18] Erler H, Ren B, Nishith G, Eric B. 2018. The intracellular parasite *Toxoplasma gondii* harbors three druggable FNT-type formate and l-lactate transporters in the plasma membrane. J Biol Chem 293:17622–17630. doi:10.1074/jbc.RA118.003801. 30237165 PMC6231131

[19] Marchetti RV, Lehane AM, Shafik SH, Winterberg M, Martin RE, Kirk K. 2015. A lactate and formate transporter in the intraerythrocytic malaria parasite, *Plasmodium falciparum*. Nat Commun 6:6721. doi:10.1038/ncomms7721. 25823844

[20] Golldack A, Henke B, Bergmann B, Wiechert M, Erler H, Blancke Soares A, Spielmann T, Beitz E. 2017. Substrate-analogous inhibitors exert antimalarial action by targeting the *Plasmodium* lactate transporter PfFNT at nanomolar scale. PLoS Pathog 13:e1006172. doi:10.1371/journal.ppat.1006172. 28178358 PMC5298233

[21] Martin RE, Henry RI, Abbey JL, Clements JD, Kirk K. 2005. The ‘permeome’ of the malaria parasite: an overview of the membrane transport proteins of *Plasmodium falciparum*. Genome Biol 6:R26. doi:10.1186/gb-2005-6-3-r26. 15774027 PMC1088945

[22] Westheimer FH. 1987. Why nature chose phosphates. Science 235:1173–1178. doi:10.1126/science.2434996. 2434996

[23] Shin H, Shin HS, Dewbre GR, Harrison MJ. 2004. Phosphate transport in *Arabidopsis*: Pht1;1 and Pht1;4 play a major role in phosphate acquisition from both low- and high-phosphate environments. Plant J 39:629–642. doi:10.1111/j.1365-313X.2004.02161.x. 15272879

[24] Versaw WK, Harrison MJ. 2002. A chloroplast phosphate transporter, PHT2;1, influences allocation of phosphate within the plant and phosphate-starvation responses. Plant Cell 14:1751–1766. doi:10.1105/tpc.002220. 12172020 PMC151463

[25] Hamel P, Saint-Georges Y, de Pinto B, Lachacinski N, Altamura N, Dujardin G. 2004. Redundancy in the function of mitochondrial phosphate transport in *Saccharomyces cerevisiae* and *Arabidopsis thaliana*. Mol Microbiol 51:307–317. doi:10.1046/j.1365-2958.2003.03810.x. 14756774

[26] Guo B, Jin Y, Wussler C, Blancaflor EB, Motes CM, Versaw WK. 2008. Functional analysis of the *Arabidopsis* PHT4 family of intracellular phosphate transporters. New Phytol 177:889–898. doi:10.1111/j.1469-8137.2007.02331.x. 18086223

[27] Liu TY, Huang TK, Yang SY, Hong YT, Huang SM, Wang FN, Chiang SF, Tsai SY, Lu WC, Chiou TJ. 2016. Identification of plant vacuolar transporters mediating phosphate storage. Nat Commun 7:11095. doi:10.1038/ncomms11095. 27029856 PMC4821872

[28] Bieleski RL. 1973. Phosphate pools, phosphate transport, and phosphate availability. Annu Rev Plant Physiol 24:225–252. doi:10.1146/annurev.pp.24.060173.001301.

[29] Xu L, Zhao H, Wan R, Liu Y, Xu Z, Tian W, Ruan W, Wang F, Deng M, Wang J, Dolan L, Luan S, Xue S, Yi K. 2019. Identification of vacuolar phosphate efflux transporters in land plants. Nat Plants 5:84–94. doi:10.1038/s41477-018-0334-3. 30626920

[30] Gutierrez-Alanis D, Ojeda-Rivera JO, Yong-Villalobos L, Cardenas-Torres L, Herrera-Estrella L. 2018. Adaptation to phosphate scarcity: tips from *Arabidopsis* roots. Trends Plant Sci 23:721–730. doi:10.1016/j.tplants.2018.04.006. 29764728

[31] Smith SE, Smith FA, Jakobsen I. 2003. Mycorrhizal fungi can dominate phosphate supply to plants irrespective of growth responses. Plant Physiol 133:16–20. doi:10.1104/pp.103.024380. 12970469 PMC1540331

[32] Wykoff DD, O’Shea EK. 2001. Phosphate transport and sensing in *Saccharomyces cerevisiae*. Genetics 159:1491–1499. doi:10.1093/genetics/159.4.1491. 11779791 PMC1450841

[33] Almaguer C, Cheng W, Nolder C, Patton-Vogt J. 2004. Glycerophosphoinositol, a novel phosphate source whose transport is regulated by multiple factors in *Saccharomyces cerevisiae*. J Biol Chem 279:31937–31942. doi:10.1074/jbc.M403648200. 15145930

[34] Saliba KJ, Martin RE, Broer A, Henry RI, McCarthy CS, Downie MJ, Allen RJ, Mullin KA, McFadden GI, Bröer S, Kirk K. 2006. Sodium-dependent uptake of inorganic phosphate by the intracellular malaria parasite. Nature 443:582–585. doi:10.1038/nature05149. 17006451

[35] Asady B, Dick CF, Ehrenman K, Sahu T, Romano JD, Coppens I. 2020. A single Na^+^-P_i_ cotransporter in *Toxoplasma* plays key roles in phosphate import and control of parasite osmoregulation. PLoS Pathog 16:e1009067. doi:10.1371/journal.ppat.1009067. 33383579 PMC7817038

[36] Sidik SM, Huet D, Ganesan SM, Huynh MH, Wang T, Nasamu AS, Thiru P, Saeij JPJ, Carruthers VB, Niles JC, Lourido S. 2016. A genome-wide CRISPR Screen in *Toxoplasma* identifies essential apicomplexan genes. Cell 166:1423–1435.e12. doi:10.1016/j.cell.2016.08.019. 27594426 PMC5017925

[37] Lamarque MH, Roques M, Kong-Hap M, Tonkin ML, Rugarabamu G, Marq JB, Penarete-Vargas DM, Boulanger MJ, Soldati-Favre D, Lebrun M. 2014. Plasticity and redundancy among AMA-RON pairs ensure host cell entry of *Toxoplasma* parasites. Nat Commun 5:4098. doi:10.1038/ncomms5098. 24934579

[38] Straub KW, Peng ED, Hajagos BE, Tyler JS, Bradley PJ. 2011. The moving junction protein RON8 facilitates firm attachment and host cell invasion in *Toxoplasma gondii*. PLoS Pathog 7:e1002007. doi:10.1371/journal.ppat.1002007. 21423671 PMC3053350

[39] Patton-Vogt JL, Henry SA. 1998. GIT1, a gene encoding a novel transporter for glycerophosphoinositol in *Saccharomyces cerevisiae*. Genetics 149:1707–1715. doi:10.1093/genetics/149.4.1707. 9691030 PMC1460278

[40] Schwartzman JD, Pfefferkorn ER. 1982. *Toxoplasma gondii*: purine synthesis and salvage in mutant host cells and parasites. Exp Parasitol 53:77–86. doi:10.1016/0014-4894(82)90094-7. 7198995

[41] Krug EC, Marr JJ, Berens RL. 1989. Purine metabolism in *Toxoplasma gondii*. J Biol Chem 264:10601–10607. doi:10.1016/S0021-9258(18)81663-5. 2732241

[42] Pawlowic MC, Somepalli M, Sateriale A, Herbert GT, Gibson AR, Cuny GD, Hedstrom L, Striepen B. 2019. Genetic ablation of purine salvage in *Cryptosporidium parvum* reveals nucleotide uptake from the host cell. Proc Natl Acad Sci USA 116:21160–21165. doi:10.1073/pnas.1908239116. 31570573 PMC6800313

[43] Zhang M, Wang C, Otto TD, Oberstaller J, Liao X, Adapa SR, Udenze K, Bronner IF, Casandra D, Mayho M, Brown J, Li S, Swanson J, Rayner JC, Jiang RHY, Adams JH. 2018. Uncovering the essential genes of the human malaria parasite *Plasmodium falciparum* by saturation mutagenesis. Science 360:eaap7847. doi:10.1126/science.aap7847. 29724925 PMC6360947

[44] Liang X, Cui J, Yang X, Xia N, Li Y, Zhao J, Gupta N, Shen B. 2020. Acquisition of exogenous fatty acids renders apicoplast-based biosynthesis dispensable in tachyzoites of *Toxoplasma*. J Biol Chem 295:7743–7752. doi:10.1074/jbc.RA120.013004. 32341123 PMC7261779

[45] Tamura K, Stecher G, Peterson D, Filipski A, Kumar S. 2013. MEGA6: Molecular Evolutionary Genetics Analysis version 6.0. Mol Biol Evol 30:2725–2729. doi:10.1093/molbev/mst197. 24132122 PMC3840312

[46] Shen B, Brown KM, Lee TD, Sibley LD. 2014. Efficient gene disruption in diverse strains of *Toxoplasma gondii* using CRISPR/CAS9. mBio 5:e01114-14. doi:10.1128/mBio.01114-14. 24825012 PMC4030483

[47] Hortua Triana MA, Marquez-Nogueras KM, Chang L, Stasic AJ, Li C, Spiegel KA, Sharma A, Li ZH, Moreno SNJ. 2018. Tagging of weakly expressed *Toxoplasma gondii* calcium-related genes with high-affinity tags. J Eukaryot Microbiol 65:709–721. doi:10.1111/jeu.12626. 29672999 PMC6175649

[48] Huynh M-H, Carruthers VB. 2009. Tagging of endogenous genes in a *Toxoplasma gondii* strain lacking Ku80. Eukaryot Cell 8:530–539. doi:10.1128/EC.00358-08. 19218426 PMC2669203

[49] Meissner M, Brecht S, Bujard H, Soldati D. 2001. Modulation of myosin A expression by a newly established tetracycline repressor-based inducible system in *Toxoplasma gondii*. Nucleic Acids Res 29:e115. doi:10.1093/nar/29.22.e115. 11713335 PMC92585

[50] Gaji RY, Johnson DE, Treeck M, Wang M, Hudmon A, Arrizabalaga G. 2015. Phosphorylation of a myosin motor by TgCDPK3 facilitates rapid initiation of motility during *Toxoplasma gondii* egress. PLoS Pathog 11:e1005268. doi:10.1371/journal.ppat.1005268. 26544049 PMC4636360

[51] Mazumdar J, Wilson EH, Masek K, Hunter CA, Striepen B. 2006. Apicoplast fatty acid synthesis is essential for organelle biogenesis and parasite survival in *Toxoplasma gondii*. Proc Natl Acad Sci USA 103:13192–13197. doi:10.1073/pnas.0603391103. 16920791 PMC1559775

[52] Love MI, Huber W, Anders S. 2014. Moderated estimation of fold change and dispersion for RNA-seq data with DESeq2. Genome Biol 15:550–570. doi:10.1186/s13059-014-0550-8. 25516281 PMC4302049

