# Supplementary material for: A Coccidia-Specific Phosphate Transporter Is Essential for the Growth of Toxoplasma gondii Parasites
Source: Microbiol Spectr. 2022 Sep 12;10(5):e02186-22. doi: 10.1128/spectrum.02186-22 (PMC9604053; doi:10.1128/spectrum.02186-22)
Supplement: Supplemental file 1 — Fig. S1 to S10. Download spectrum.02186-22-s0001.pdf, PDF file, 1.0 MB [file spectrum.02186-22-s0001.pdf]

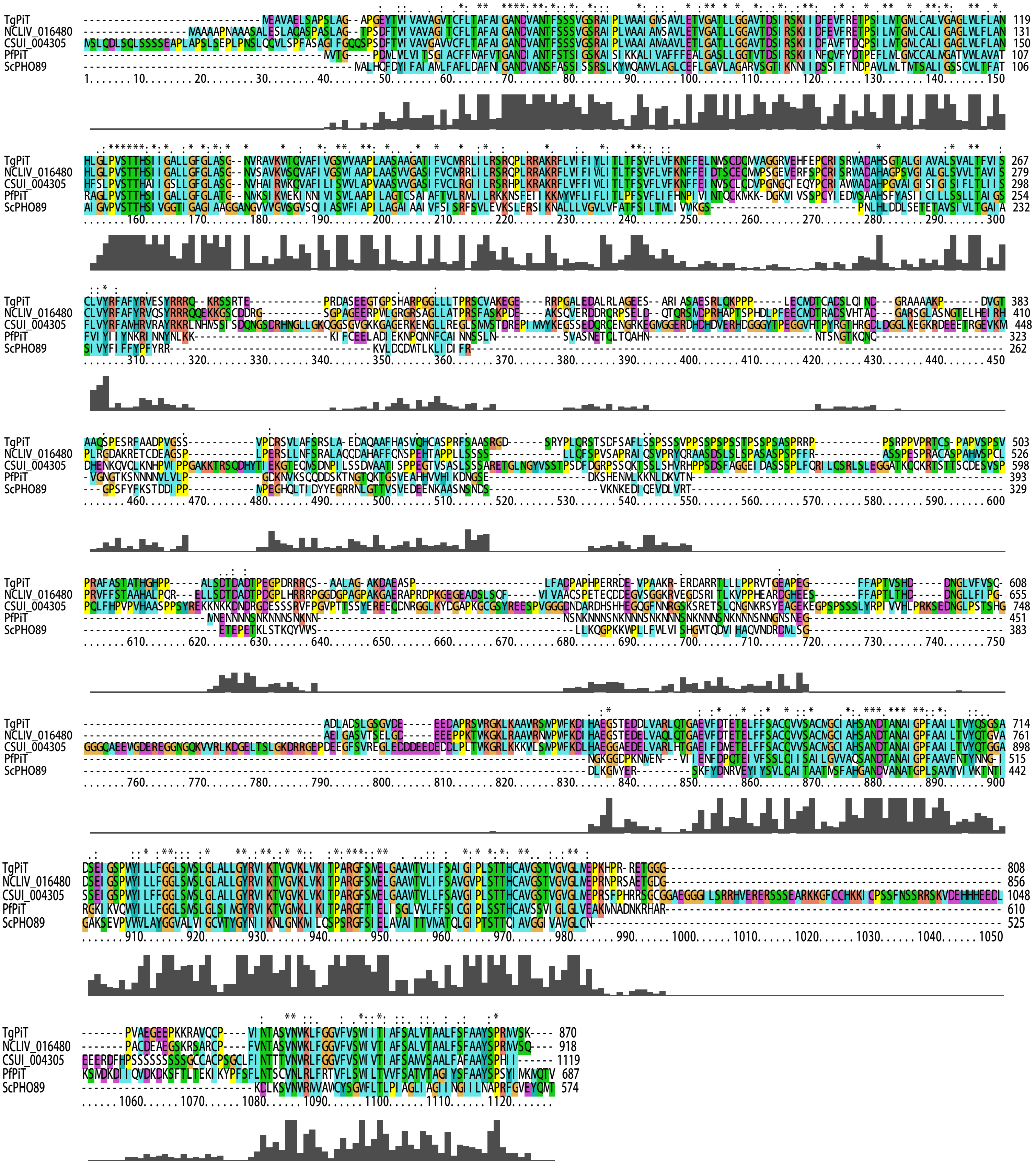

FIG S1 Sequence alignment of TgPiT and related proteins. Sequence alignment was performed in Clustal X2. TgPiT (ToxoDB: TGGT1_240210): *Toxoplasma gondii* PiT ; NCLIV_016480 (ToxoDB: NCLIV_016480): *Neospora caninum* Liverpool PiT; CSUI_004305 (ToxoDB: CSUI_004305): *Cystoisospora suis* PiT; PfPiT (PlasmoDB: PF3D7_1340900): *Plasmodium falciparum* PiT; ScPHO89 (NCBI: KZV13384.1): *Saccharomyces cerevisiae* PHO89.

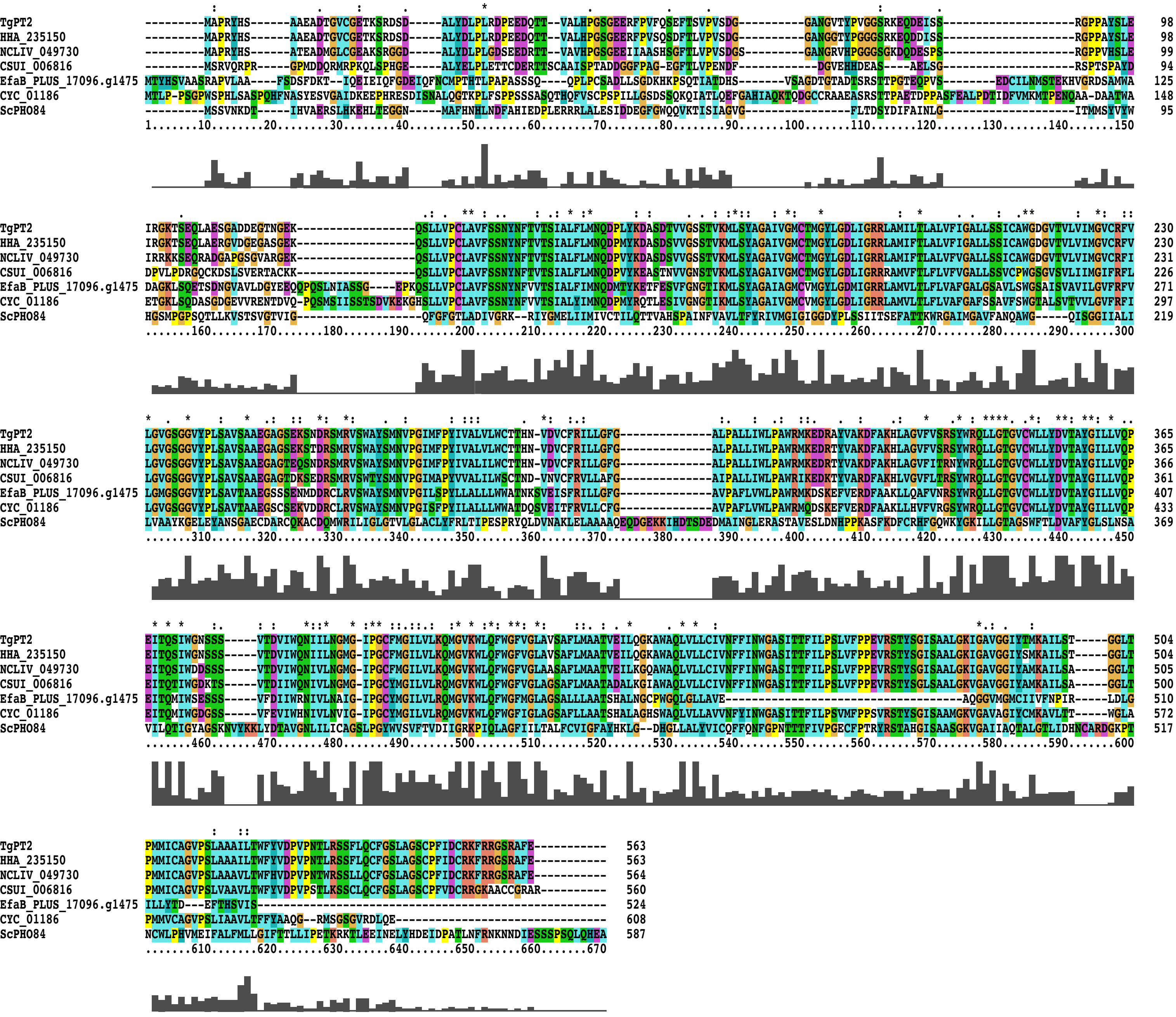

FIG S2 Sequence alignment of TgPT2 and related proteins. Sequence alignment was performed in Clustal X2. TgPT2 (ToxoDB: TGGT1_235150): *Toxoplasma gondii* PT2; HHA_235150 (ToxoDB: HHA_235150): *Hammondia hammondi* PT2; NCLIV_049730 (ToxoDB: NCLIV_049730): *Neospora caninum* Liverpool PT2; CSUI_006816_t36 (ToxoDB: CSUI_006816): *Cystoisospora suis* PT2; EfaB_PLUS_17096.g1475 (ToxoDB: EfaB_PLUS_17096.g1475): *Eimeria falciformis* PT2; CYC_01186 (ToxoDB: CYC_01186): *Cyclospora cayetanensis* PT2; ScPHO84 (NCBI: KZV08715.1): *Saccharomyces cerevisiae* PHO84.

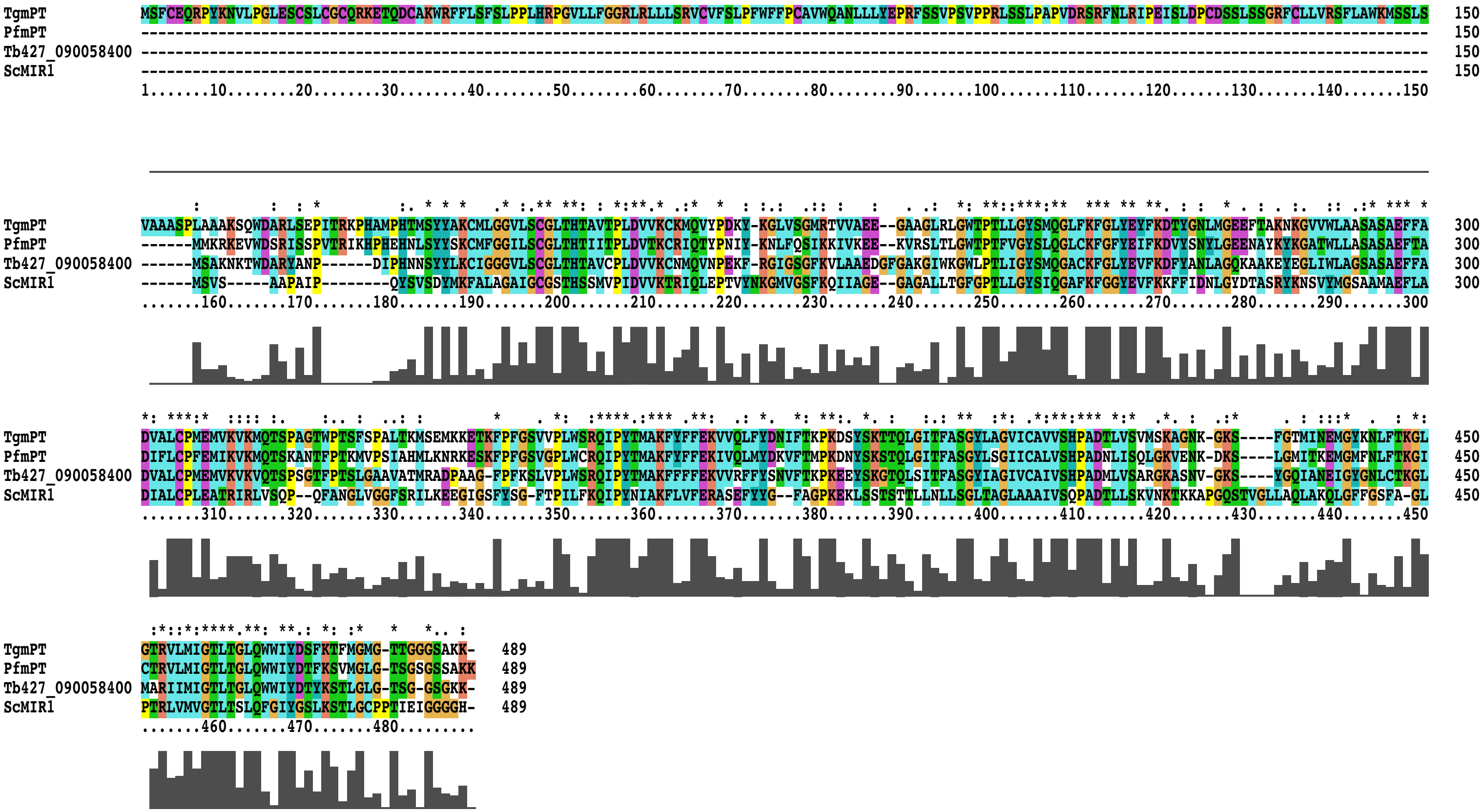

FIG S3 Sequence alignment of TgmPT and related proteins. Sequence alignment was performed in Clustal X2. TgmPT (ToxoDB: TGGT1_278990): *Toxoplasma gondii* mPT; PfmPT (PlasmoDB: PF3D7_1202200): *Plasmodium falciparum* mPT; Tb427_090058400 (TritrypDB: Tb427_090058400): *Trypanosoma brucei* mPT; ScMIR1 (NCBI: CAA89605.1): *Saccharomyces cerevisiae* mPT.

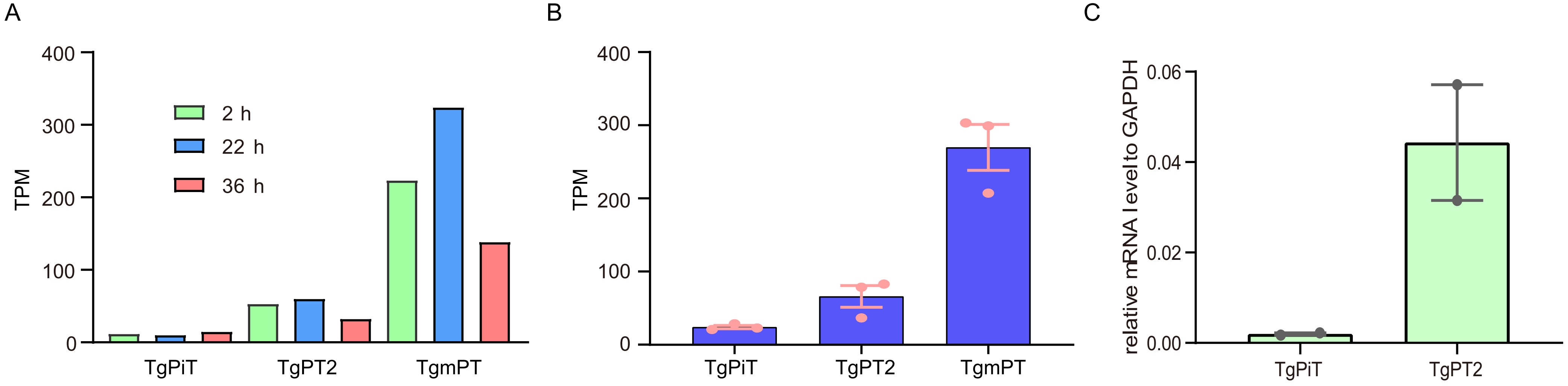

FIG S4 Expression levels of phosphate transporter encoding genes in *Toxoplasma*. (A) Transcript levels of the corresponding genes in the RH strain 2 h, 22 h or 36 h after invasion into HFF cells, respectively. The data were derived from ToxoDB. (B) The transcript level of each phosphate transporter in RH parasites, derived from the RNA-seq data obtained in this study. n = 3. (C) qRT-PCR analysis of mRNA level of TgPiT and TgPT2 in RH parasites, n=2. Mean ± SEM; * $p < 0.05$, ** $p < 0.01$. Unpaired two-tailed Student's t-test.

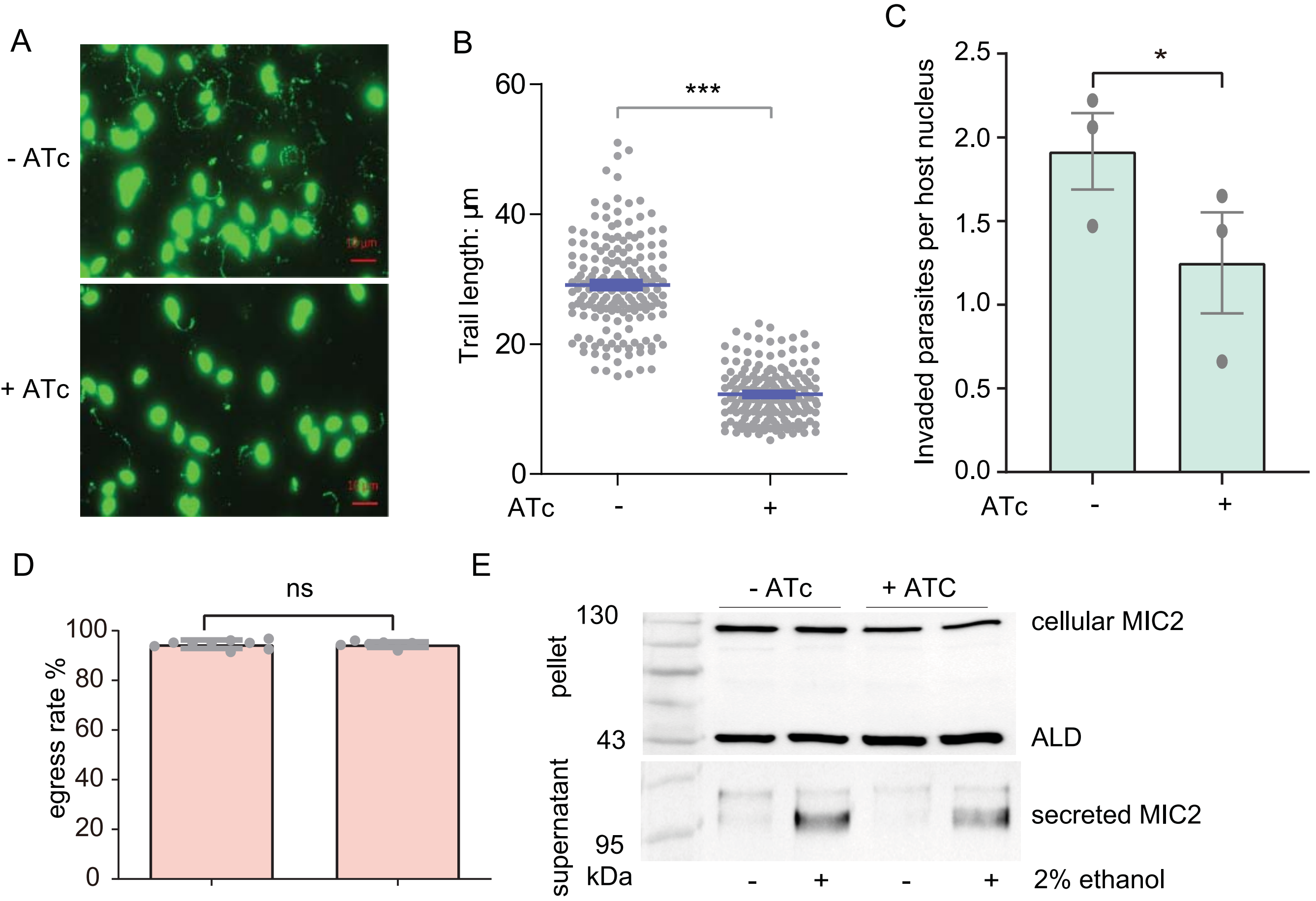

FIG S5 Reduced motility and host-cell invasion efficiency of the TgPT2 depletion mutants. (A) Representative images of parasite motility on BSA coated coverslips. The iKD-PT2 strain was treated with or without ATc for 48 hours and then freshly egressed parasites were added to coverslips to initiate gliding motility, which was visualized by SAG1 staining. (B) Trail length recorded from experiments described in A. (C) Host cell invasion efficiency of the iKD-PT2 strain treated with or without ATc. (D) Egress rates of iKD-PT2 parasites treated with or without ATc for 40 hours. Egress was induced by A23187 at final concentration of 2 μM. (E) Western blot to assess the effect of TgPT2 suppression (by ATc treatment for 48 hours of the iKD-PT2 strain) on microneme secretion using MIC2 discharge as a reporter. Microneme secretion was induced in serum free DMEM with 2% ethanol. The supernatant was separated from intact parasites by centrifugation. TgMIC2 and TgALD, which was used as loading control, were detected by mAb 6D10 and rabbit anti-TgALD, respectively. Mean ± SEM;****p* < 0.001, **p*< 0.05 (n=3 assays). Paired two-tailed student's *t*-test.

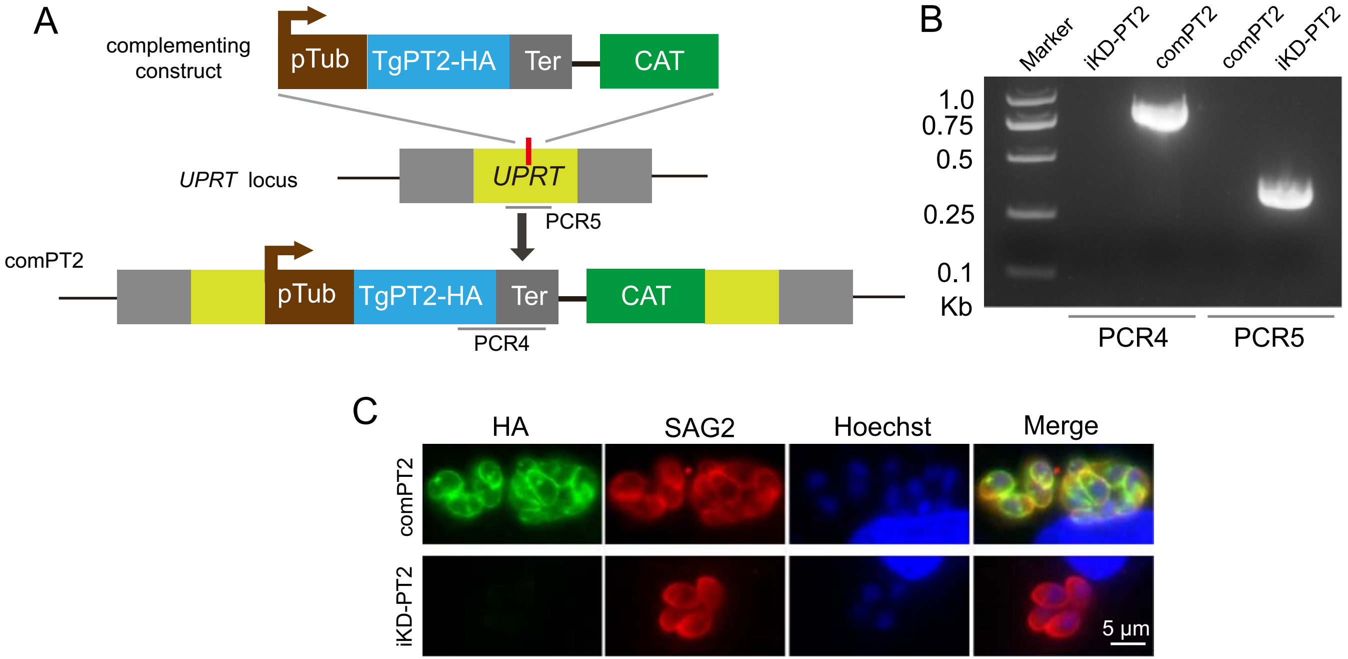

FIG S6 Construction of a TgPT2 complementing strain. (A) schematic illustration of inserting a TgPT2 (HA tagged) expressing cassette into the *UPRT* locus of the iKD-PT2 strain to construct the comPT2 strain for complementation. Red bar indicates the CRISPR targeting site in *UPRT*. PCR4/5 are used to identify the correct insertion of TgPT2. (B) Diagnostic PCR of one comPT2 clone. (C) immunofluorescent assay to examine the expression of complementing PT2 in the comPT2 clone.

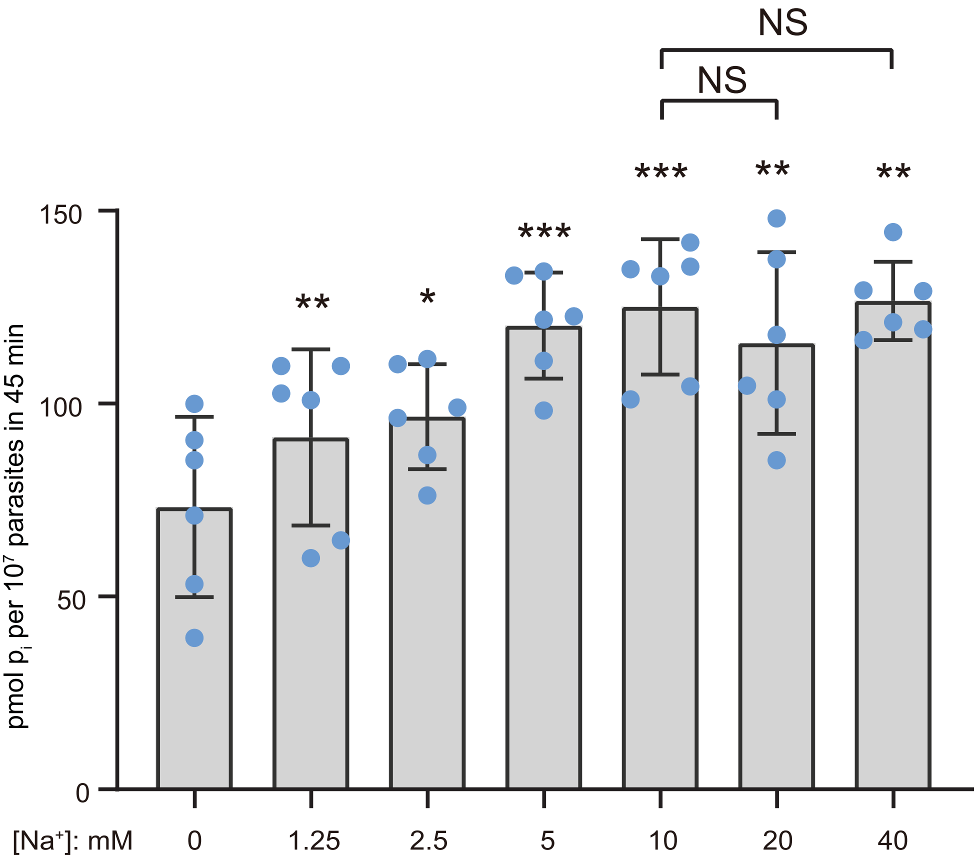

FIG S7 Effect of Na^+ concentration on P_i uptake in TATi parasites. The assays were done in the Na^+ dependent Pi transport buffer containing different Na^+ concentrations, and choline chloride was used to balance the osmolarity. Mean ± SEM; n= 6, * $p < 0.05$, ** $p < 0.01$, *** $p < 0.001$, compared with the $[Na^+] = 0$ group. NS: not significantly different, paired two-tailed Student's t-test.

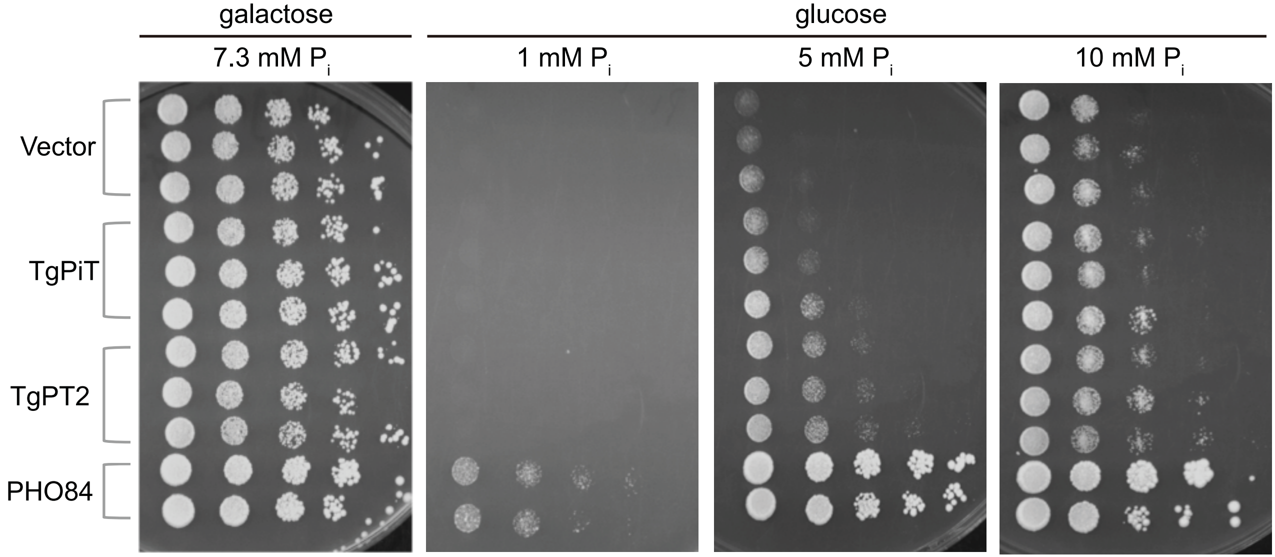

FIG S8 Evaluation of the P_i transport activity of *Toxoplasma* phosphate transporters in yeasts. Complementation of the yeast mutant YP100 (*Δpho84 Δpho87 Δpho89 Δpho90 Δpho91 Δgit1 pGal::pho84*) defective in P_i transport by TgPiT or TgPT2. Five-microliter aliquotes of 5-fold serial dilutions of each strain were spotted onto agar plates made from P_i free YNB (pH 5.5) medium containing different P_i concentrations, respectively. The plates were incubated at 30℃ for 5 days before imaging. Complementing the same mutant with the empty vector or yeast PHO84 was used as negative and positive controls respectively. In the presence of galactose, expression of pho84 from *pGal::pho84* was active and all strains grew equally well. However, in the presence of glucose (no galactose), expression of pho84 from *pGal::pho84* was supressed and growth of yeasts depended on the complementing transporters and P_i concentrations.

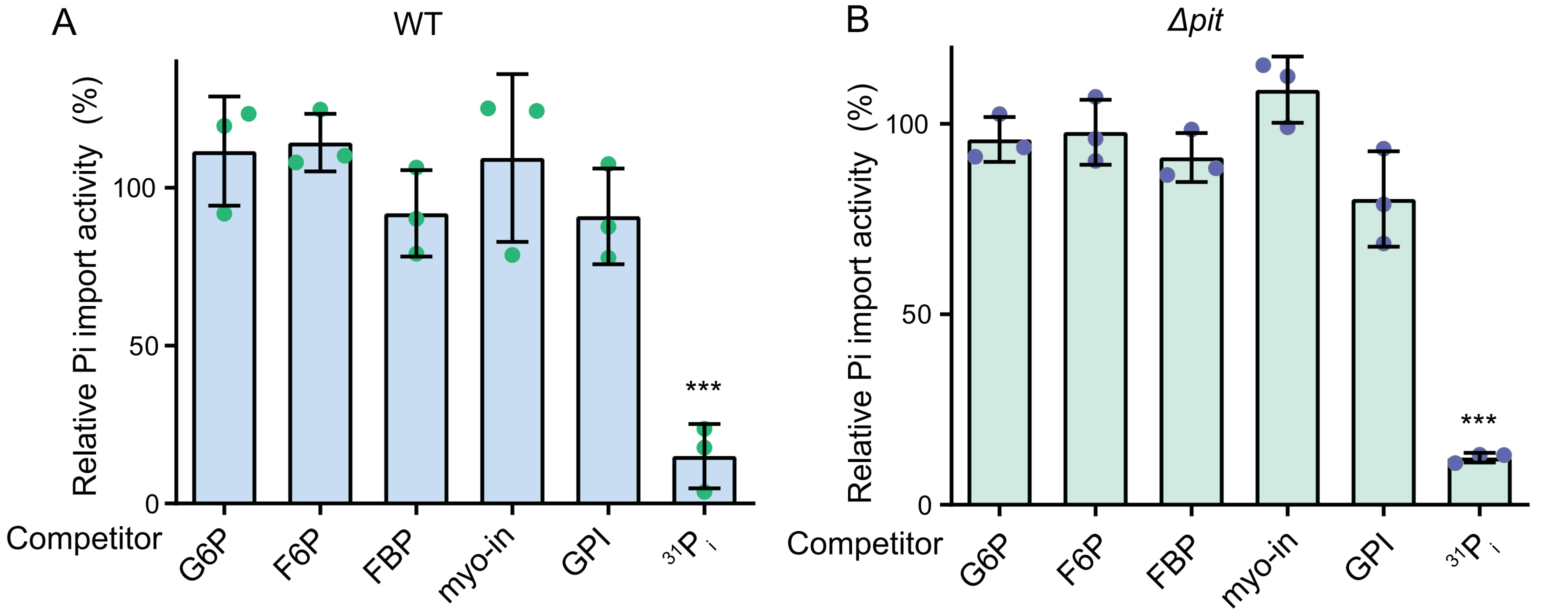

FIG S9 Competitive inhibition of P_i uptake by organic compounds. Purified tachyzoites of the wildtype (A) and *Δpit* (B) strains were used in a 45-minutes P_i uptake assay as described in **FIG 5** (assay conditions: P_i free DMEM, pH = 7.4,), in the presence of 1 mM glucose-6-phosphate (G6P), 1mM fructose-6-phosphate (F6P), 1mM fructose-1,6-bisphosphate (FBP), 250 μM myo-inositol (myo-in) or 100 μM glycerophosphoinositol (GPI). 1 mM inorganic phosphate (P_i) was used as a positive control. Transport activity with no competitor was set at 100% and used as a normalization reference for the rest conditions. Each compound was tested three times independently. Mean ± SEM; ***p < 0.001 (n=3 assays). Paired two-tailed student's *t*-test, compared with the no competition control.

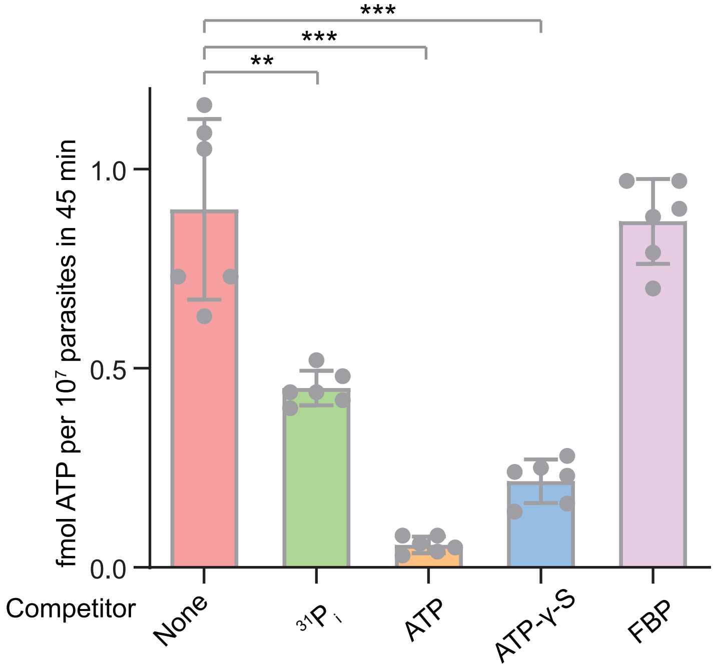

Fig S10 Uptake of ATP by *Toxoplasma* parasites. The wild type RH parasites were collected to test their ATP uptake activity using 4 μCi /ml ATP-α-P^{32}. Meanwhile, 1 mM non-radioactive KH_2PO_4, ATP, ATP-γ-S or FBP was included in the reaction system as competitors. Mean ± SEM; n=3. ** $p < 0.01$, *** $p < 0.001$, Paired two-tailed Student's *t*-test. Note that the calculated amounts of ATP imported are quite different from that obtained in Fig 6C (the no competitor group in both figures). This is likely caused by the different efficiency of P^{32} labeling in the two products (ATP-α-P^{32} vs ATP-γ-P^{32}) because we detected different levels of radioactivity even if the same amount (in pmol) of radioactive ATP was added.
